# Integrated transcriptome analysis of mouse spermatogenesis

**DOI:** 10.1186/1471-2164-15-39

**Published:** 2014-01-18

**Authors:** Gennady Margolin, Pavel P Khil, Joongbaek Kim, Marina A Bellani, R Daniel Camerini-Otero

**Affiliations:** 1 Genetics and Biochemistry Branch, National Institute of Diabetes and Digestive and Kidney Diseases (NIDDK), National Institutes of Health (NIH), Building 5, Room 205A, Bethesda, MD 20892, USA; 2 National Institute of Aging, National Institutes of Health (NIH), Baltimore, MD 21224, USA

**Keywords:** Spermatogenesis, Meiosis, RNA-Seq, Transcriptome, Deconvolution, RNA Pol II, piRNA

## Abstract

**Background:**

Differentiation of primordial germ cells into mature spermatozoa proceeds through multiple stages, one of the most important of which is meiosis. Meiotic recombination is in turn a key part of meiosis. To achieve the highly specialized and diverse functions necessary for the successful completion of meiosis and the generation of spermatozoa thousands of genes are coordinately regulated through spermatogenesis. A complete and unbiased characterization of the transcriptome dynamics of spermatogenesis is, however, still lacking.

**Results:**

In order to characterize gene expression during spermatogenesis we sequenced eight mRNA samples from testes of juvenile mice from 6 to 38 days post partum. Using gene expression clustering we defined over 1,000 novel meiotically-expressed genes. We also developed a computational de-convolution approach and used it to estimate cell type-specific gene expression in pre-meiotic, meiotic and post-meiotic cells. In addition, we detected 13,000 novel alternative splicing events around 40% of which preserve an open reading frame, and found experimental support for 159 computational gene predictions. A comparison of RNA polymerase II (Pol II) ChIP-Seq signals with RNA-Seq coverage shows that gene expression correlates well with Pol II signals, both at promoters and along the gene body. However, we observe numerous instances of non-canonical promoter usage, as well as intergenic Pol II peaks that potentially delineate unannotated promoters, enhancers or small RNA clusters.

**Conclusions:**

Here we provide a comprehensive analysis of gene expression throughout mouse meiosis and spermatogenesis. Importantly, we find over a thousand of novel meiotic genes and over 5,000 novel potentially coding isoforms. These data should be a valuable resource for future studies of meiosis and spermatogenesis in mammals.

## Background

**Figure 1:**
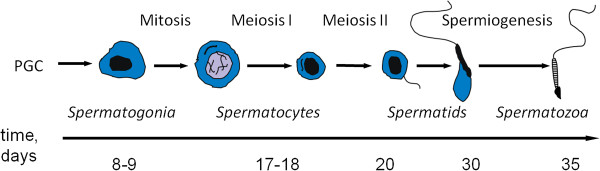
**A time course of spermatogenesis.** Primordial germ cells (PGC’s) are the germline progenitors. They give rise to various types of spermatogonia, which proliferate and differentiate into spermatocytes. Meiosis and genetic recombination occur in spermatocytes, which become haploid spermatids after the completion of meiosis. Spermatids undergo the process of spermiogenesis until their maturity, which includes elongation, genome condensation and formation of a flagellum. The approximate timeline of the first wave of spermatogenesis in mice, in days after birth (dpp) is indicated. The figure is adopted from [45] by the authors holding the copyright.

Spermatogenesis is a complex multistage process involving thousands of genes, and it is especially difficult to study in mammals [1-3]. In essence it is the process in males by which diploid cells give rise to haploid gametes. Briefly, germline cells are derived from primordial germ cells (PGC), that give rise to primitive spermatogonia A (pSGA). Some daughter cells of mitotically dividing pSGAs differentiate into more advanced spermatogonia A subtypes, which eventually give rise to spermatogonia B and proceed through the stages of meiotic spermatocytes into spermatids and mature sperm [1,4,5]. Meiosis is central to gametogenesis, and in male mice it starts about 8 days post partum (dpp). The outcome of meiosis of a single spermatogonium is four haploid spermatids. Meiosis lasts about two weeks in mice and consists of two divisions, I and II. The primary spermatocytes replicate their maternal and paternal chromosomes and then, in meiosis I, undergo a unique process in which the homologous parental chromosomes recombine with each other. Recombination generates genetic diversity and ensures the proper segregation of chromosomes. Errors in recombination can lead to either too few or too many chromosomes in the spermatids, a phenomenon referred to as aneuploidy. The secondary spermatocytes divide further in meiosis II which is comparatively very brief, a matter of hours, and is similar to a mitotic division. Meiosis II yields haploid spermatids which proceed through spermiogenesis resulting in sperm (Figure 1). Recombination of homologous chromosomes takes place during prophase of meiosis I, which consists of five stages: leptotene, zygotene, pachytene, diplotene and diakinesis, in that order, the longest of which is pachytene. In the first wave of spermatogenesis, a relatively synchronous first cycle of sperm production after birth, pachytene begins at around 12-14 dpp. During this wave, the proportions of the various cell types in the testes change over time, while consecutive waves of spermatogenesis gradually become desynchronized. The process of germ cell development is directly assisted by various somatic cells present in testes. Despite the critical importance of spermatogenesis, its complex developmental program and its accompanying changes in gene expression are still not fully understood.

Two alternative approaches have been used to study gene expression during spermatogenesis. One uses cell sorting to separate and characterize the various cell types [6,7]. Another is based on examining gene expression in the different cell populations present throughout the first wave of spermatogenesis [8,9]. Both of these sets of studies have used microarrays. Until recently, high-throughput RNA sequencing approaches have been used to characterize spermatogenesis in a much more restricted way focusing either on a single developmental time point [10,11] or on comparisons between pairs of neighboring time points [12]. A recent paper [13] utilized RNA-Seq of sorted cells to study the transcriptome of mouse testes. RNA sequencing has several important advantages compared to microarrays – better sensitivity, a greater dynamic range and the ability to detect every expressed gene or splicing variant/isoform, even if previously unknown [14].

Here we analyzed gene expression during first wave of spermatogenesis in murine pups using RNA-Seq in order to discover novel genes and isoforms active in spermatogenesis and meiosis. We analyzed testis samples from 6 to 38 day old mice with two-day sampling intervals between 10 and 20 dpp to improve the coverage of meiotic samples. We classified gene expression profiles and compared our results to previous microarray-based studies [6-9]. This comparison allowed us to identify genes that were not previously described as meiotically-expressed in high-throughput studies. We then developed a deconvolution algorithm to computationally determine cell type-specific gene expression and estimated gene expression levels in somatic cells, pre-meiotic spermatogonia, spermatocytes and spermatozoa. We validated our predictions by comparing them to the experimentally derived measurements of mRNA levels in cell-sorted samples from whole testis [13]. RNA-seq data were further mined to describe alternative splicing patterns and alternative polyadenylation site usage during spermatogenesis. Using our RNA-seq data we further evaluated computationally predicted gene models. Finally, we measured the genome-wide distribution of RNA Pol II (Pol II) at two different time points and compared it with gene expression in spermatogenesis.

## Results

### RNA-seq of mouse spermatogenesis

In order to study gene expression during spermatogenesis, we sequenced mRNA samples from whole testes of pre-pubertal mice at 10, 12, 14, 16, 18 and 20 days post partum (dpp) which include the meiotic stages of the first wave of spermatogenesis. We also analyzed pre-meiotic (6 dpp) and adult (38 dpp) samples. We generated between 42 and 96 millions of reads for each sample (Additional file 1: Table S1). There were between 5.5 and 9.8 million genomic locations with uniquely aligned reads (Additional file 1: Table S1). To estimate the quality of our protocol of mRNA preparation and sequencing, we plotted the average read coverage of all genes relative to the position within the transcript (Additional file 1: Figure S1). These distributions are mostly even for all samples indicating that the RNA samples were subjected to limited degradation.

Overall, we find that around 85% of protein coding genes might be expressed at each time point (that is, have at least one mapped read). Such a high proportion of expressed genes may be at least in part explained by the presence of many cell types in our samples [15-17]. A stricter criterion of gene expression is the observation of exon splicing: in each sample, we see spliced reads in over 50% of protein-coding genes (Additional file 1: Table S2). Although the vast majority of genes are expressed in our samples, 1,008 protein coding genes were not detected in any of the samples. Olfactory receptor (OLFR) genes were highly enriched among those non-expressed genes (p-value <4∙10^-150^). Although some olfactory receptor genes are expressed in various tissues including testis, most of the members of the superfamily are expressed only in olfactory sensory neurons [18,19]. Thus, the observed enrichment of OLFR genes is consistent with the neuronally-restricted expression pattern of the majority of olfactory receptors.

### Temporal expression clusters

**Figure 2:**
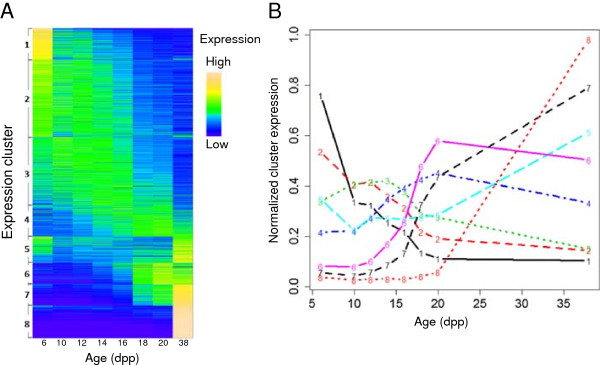
**Temporal gene expression clustering.** Genes with maximal expression above 2 RPKM, at least two-fold expression change and a mature transcript length ≥100b were clustered. Given these criteria, 12,895 genes were selected for clustering into 8 clusters. The cluster sizes are 1313, 3227, 2826, 1302, 1087, 900, 895 and 1345 for clusters from 1 through 8, respectively. The heatmap **(A)** and normalized expression profiles of cluster centroids **(B)** are shown. As clusters 3, 4, 6 and 7 show a rising profile during meiosis, 8 to 20dpp, these clusters were designated as meiotic clusters.

To functionally characterize the global gene expression patterns we clustered the gene expression profiles using k-means. We only considered genes with a sufficiently high expression level in at least one of the 8 samples (>2 Reads per Kilobase per Million of Reads, RPKM), which change their expression by at least 2-fold, and have an mRNA transcript length of at least 100 bases. As a first approximation, for most genes with varying expression their expression either decreases or increases monotonically with time (Figure 2). One group of genes (cluster 8) is mainly expressed only in the adult sample, indicating that these genes turn on late in spermatogenesis. The expression of the majority of the clustered genes decreases with age (dpp). However, some genes for which expression peaks in the middle of the time course are also discernible. To have a finer resolution of gene profiles, we split them into 8 clusters (Figure 2; see Materials and Methods and Additional file 2).

Next, we compared our clustering with three previously published papers – Chalmel et al. [6], Shima et al. [9] and Schultz et al. [8]. Overall we find a good agreement - 89% or more genes present in common are classified similarly (Additional file 1: Figure S2). To further validate our clustering results we looked at several well known genes associated with meiosis and their corresponding clusters in the present study, as well as in the three microarray studies discussed above (Table 1; see also Additional file 1: Table S3 for an extended list of genes assigned with Gene Ontology terms meiosis and spermatogenesis). While there is an overall agreement for genes interrogated by both platforms, there are some potentially misclassified genes in the microarray studies. For example, *Dmc1* is in the post-meiotic (PM) cluster of Chalmel et al., while *Mnd1* is in the early expression cluster A of Shima et al. In fact, *Dmc1* and *Mnd1* genes play important roles during meiotic recombination and belong to our intermediate cluster 3 (one of our meiotic clusters, see below). In agreement with our clustering, immunohistochemical analysis of *Dmc1* protein found it in leptotene-to-zygotene spermatocytes [20]. Another example is *Prdm9* gene, which has recently attracted much attention due to its role in determining meiotic recombination [21-23]. There are no probe sets for this gene in the Affymetrix microarrays used in [8] and [9], and it was not classified in [6], probably due to a lack of a signal. Similarly, the recently characterized gene *Spata22*[24], known to be essential for meiotic progression, is also absent from the microarrays.

**Table 1:** Expression profiles of selected genes associated with meiosis

**Gene**	**Temporal 1-8**	**Schultz’03 1-8**	**Shima’04 A-E**	**Chalmel’07 SO,MI,ME,PM**	**Deconvolution A-E**
***Aym1***	6	5	D	ME	D
***Brca1***	2	2	-	-	B
***Brca2***	3	6	-	-	B
***Dazl***	3	4	-	ME	B
***Dmc1***	3	4	-	PM	B
***Fancm***^§^	3	-	-	-	B
***Itga6***	1	2	-	MI	A
***Itgb1***	2	2	A	SO, MI	B
***Mei1***^§^	3	-	-	-	B
***Mei4***^§^	3	-	-	-	-
***Mlh1***^§^	3	-	-	-	C
***Mnd1***	3	3	A	-	-
***Mre11a***^§^	Const	-	-	-	C
***Msh4***	4	6	D	PM	C
***Nbn***	Const	2	-	-	B
***Pms2***	3	3	C	-	C
***Prdm9***^§^	3	-	-	-	B
***Psmc3ip***	6	7	D	ME	C
***Rad21***	2	-	-	MI	B
***Rad50***	3	2	B	-	B
***Rad51***	3	3	-	MI	B
***Rad51c***	Const	5	D	ME	D
***Rad51l1***	Low	6	-	-	-
***Rad51l3***^§^	3	-	-	-	C
***Rad52***	3	6	D	-	C
***Rad54l***^§^	3	-	-	-	B
***Rec8***	5	5	E	PM	E
***Rpa1***	5	5	D	-	D
***Spag4l***	8	8	E	PM	E
***Spata22***^§^	4	-	-	-	C
***Spo11***	6	5	D	ME	C
***Stra8***	3	4	C	MI	B
***Sun1***	Const	-	-	-	C
***Sycp1***	4	4	C	ME	C
***Sycp2***	4	-	-	ME	C
***Sycp3***	4	-	-	ME	C
***Tex11***	3	-	-	MI	B

While *Dmc1* and *Mnd1* are found in our cluster 3 – see Figure 2 – they are either misclassified, or unclassified by the microarray-based study of Shima et al. [9] and Chalmel et al. [6]. Note that there were no Affimetrix probes in the regions of the *Spata22* gene and *Prdm9* gene (in [8] and [9]). Genes marked with (^§^) have not been either interrogated or classified in the microarray studies [6,8,9].

Henceforth, we identify clusters 3, 4, 6 and 7 (Figure 2 and Additional file 1: Figure S2) as meiotic clusters, given that the expression levels of genes belonging to these clusters rise coincident with the appearance of various meiotic cell types. We find that in these meiotic clusters (Figure 2) there are a total of 5,923 genes. Out of these 5,923 genes nearly a quarter (1,555 total/1,048 protein coding genes) were either not present on microarrays or were not differentially expressed in previous microarray studies [6,8,9] (Additional file 3). We thus refer to these genes as novel meiotic genes. We must clarify, however, that together with more than 200 uncharacterized or poorly characterized genes, some genes with solidly established meiotic function but that were not characterized by microarrays are on this list. These genes include *Prdm9*, *Spata22*, *Morc4*, *Mei1* and *Mlh1*. To avoid any ambiguity we emphasize that our “novel meiotic genes” do not have to be expressed in testis exclusively and include some genes with previously assigned meiotic function that were not previously characterized as meiotic in other high throughput studies.

### *In silico* determination of cell type-specific gene expression

**Figure 3:**
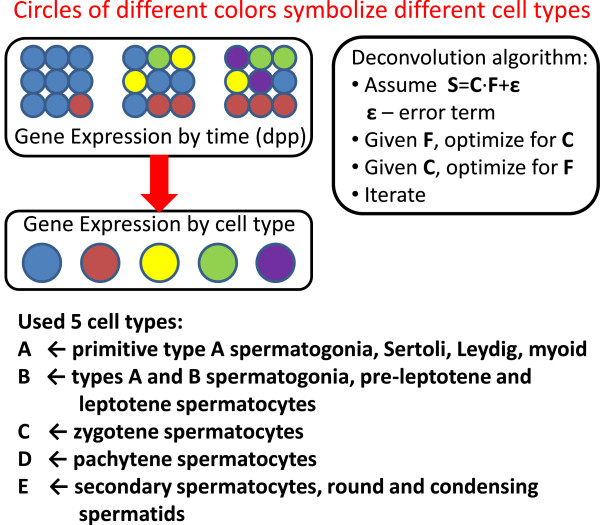
**Schematics of the deconvolution algorithm to estimate cell type-specific gene expression.** We have measured gene expression by dpp (S), and have estimates of cell type fractions by dpp from the literature (F). Our goal is to estimate gene expression by cell type (C), as well as to re-estimate cell type fractions, or cell type contributions to gene expression (F). The iterative procedure is depicted on the figure, and the details are in Materials and Methods. Due to both biological and mathematical considerations, 5 combined cell types were considered in our analysis.

Our gene expression data set is temporal – we have measurements of gene expression levels in whole mouse testis at different ages. Testes consist of somatic and pre-meiotic germ cells, meiotic spermatocytes and post-meiotic spermatids and each of these cell types contains numerous subtypes that have their own characteristic gene expression profiles [1,25]. Thus, the observed gene expression level in a sample prepared from a total testis is a sum of gene expression levels from individual cell types. Moreover, during the first wave of spermatogenesis, the proportions of different cell types change drastically. To better understand functional processes during the course of spermatogenesis it would be desirable to obtain estimates of cell type-specific gene expression. Here we use a computational approach to deconvolve temporal gene expression profiles from a mixture of cell types into cell-type specific expression profiles (Figure 3). A similar approach has been proposed and tested in the literature [26-31], although typically with fewer cell types and for microarrays.

**Figure 4:**
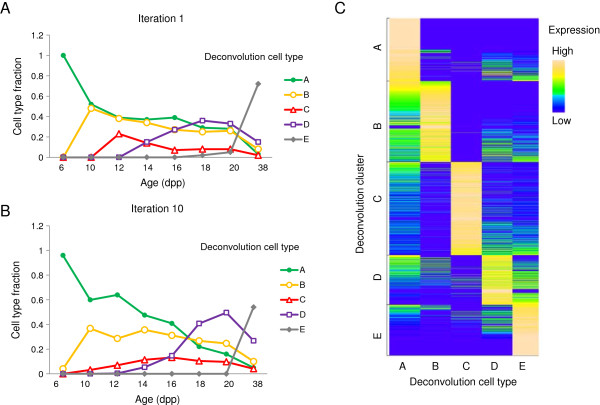
**Results of the deconvolution algorithm to estimate cell type-specific gene expression.** (Left) Cell type fractions used in the first iteration (**A**; based on Bellve et al. [25]) and obtained after 10 iterations **(B)**. **(C)** Cell type-expression heatmap of genes selected for deconvolution, obtained after 10 iterations after genes were clustered in 5 clusters, corresponding to the cell types considered. Somatic expression (cell type A) is more ubiquitous, and cell types D and E share many expressed genes.

We took advantage of the digital nature of RNA-Seq data, and developed a weighted least squares optimization algorithm that allowed us to estimate gene expression levels in individual cell types (Materials and Methods). Briefly, starting with initial estimates of cell type proportions, we estimate cell type-specific gene expression, which in turn can be used to iteratively re-estimate cell type proportions. The initial estimate of cell type fractions is based on previously reported values [32] with some of the cell types grouped together (Figure 3). Based on mathematical, as well as biological considerations, we chose to divide all cells into five cell types (or cell type groups) A through E (Materials and Methods). The fraction of non-meiotic cells (denoted A) drops significantly from 6 dpp to adult mice, while proportions of different germ cell populations rise and decay throughout the time course (Figure 4). Although there were no zygotene spermatocytes at 10 dpp in our initial estimate, they appear after 10 iterations, which is consistent with previously published experimental data [33]. Similarly, we also found that the contribution of spermatids (fraction E) to the expression in whole testis is negligible at and before 20 dpp. Similar to the clustering of temporal gene expression, we also clustered cell type-specific gene expression (Table 1). This clustering associates genes with certain cell types, similar to the classification of cell-sorted gene expression in [6] (Figure 4C and Additional file 4).

The summary of cell type-specific gene expression obtained after 10 iterations can also be represented as a heatmap (Figure 4) that shows that most genes are expressed in one or two cell types. There are many genes that are expressed both in somatic, A, and in one of the germ cell types. Also, there is a significant overlap in gene expression between pachytene and secondary spermatocytes and spermatids, D and E. This observation, which is based on a purely computational analysis of temporal expression data, is in agreement with previous experimental observations [7,34,35].

Furthermore, we have compared our cell type-specific gene expression predictions to a recently published study [13] that measured gene expression of sorted Sertoli and germ cells using RNA-Seq. There is a good correlation between the experimental measurements of gene expression reported in [13] and our deconvolution estimates (Additional file 1: Figure S3). We have clustered the reported experimental gene expression into 5 clusters, each corresponding to one of the cell types considered in [13]. This produced an overall consistent gene classification between our clusters A through E and the five clusters we derived from [13]. Drawn as a heatmap, gene expression from [13] resembles the pattern of our cell-type expression heatmap (compare Figure 4 and Additional file 1: Figure S3).

Soumillon et al. [13] also define four expression clusters – cluster 1 has high gene expression in spermatocytes and spermatids, relative to spermatogonia and spermatozoa, and cluster 2 is the opposite; cluster 3 is high in spermatogonia and spermatids versus spermatocytes and, to a lesser extent, spermatozoa; cluster 4 is low in spermatozoa relative to the three other types. Sertoli cells are not shown in this clustering. A comparison of the genes shared in our deconvolution clusters A-E and those in these four clusters shows that cluster 1 mostly corresponds to our clusters D and E, cluster 2 to our clusters A and B, and clusters 1 and 2 mostly correspond to our cluster C. Clusters 3 and 4 share very few genes with any of our clusters, because many genes in these clusters from [13] have low levels of expression. This can also be seen in the comparisons of our temporal clusters with those of [13] (Additional file 1: Figure S3, bottom).

Looking at cell type-specific gene expression (Figure 4, Additional file 1: Figure S4), we found that more genes are expressed in somatic A, while fewer genes are expressed in germ cell types (between 4,465 in C and 7,609 in E as compared to 12,300 in A, out of 14,259 considered). The median expression is largest in C – 76 RPKM vs. 15-28 RPKM in other cell types. In addition, there are 1294, 133, 107, 163 and 528 genes in cell types A, B, C, D and E, respectively, that are classified as expressed exclusively in those cell types.

While our statement that fewer genes are expressed in individual germ cell types than in somatic type A seems to disagree with the statements in [13] that more genes are expressed in germ cell types than in Sertoli cells, there are several factors to consider. First, our type A includes not only Sertoli cells (Figure 3); second, we only considered a subset of genes for the deconvolution procedure; third, the deconvolution procedure attempts to minimize the number of expressed genes needed to explain the observed temporal expression data, and is conservative in that sense; fourth, the experimental cell sorting purity is on average around 90% and so gene expression from other cell types might be observed – hence, cell sorting is a permissive approach. Our finding that the median expression is largest in C (zygotene spermatocytes) resembles the high per cell RNA count in spermatocytes reported in [13].

Using the deconvolution results, we can associate temporal clusters with specific cell types. While this could in principle be done based on the timeline of spermatogenesis, here we have a quantification of this correspondence. For example, temporal cluster 3 has the highest contribution from B (spermatogonia A and B, and pre-leptotene and leptotene spermatocytes) while temporal cluster 6 is dominated by D (pachytene spermatocytes) (see Additional file 1: Figure S5).

One way to validate our predictions is to look at some well-known genes. We found that, overall, these genes have been properly classified (Table 1, Additional file 1: Table S4). For example, the synaptonemal complex genes *Sycp1-3* were mostly found expressed in zygotene (C), protamines are expressed in spermatids (E), *Dmc1* was found in pre-leptotene and leptotene primary spermatocytes, and many Sertoli cell markers were correctly classified in cluster A (e.g., *Gdnf*, *Etv5*[36]). Spermatogonia genes (e.g., *Dazl*[36]) were classified correctly as well. Also, as cell type A includes primitive type A spermatogonia, the assignment of *Cdh1* to cluster A is appropriate [36]. Further details are discussed in Materials and Methods.

### Alternative splicing

One of the advantages of mRNA-seq over microarrays is that all expressed targets can be assessed. We were interested in finding novel exon-exon splice junctions, which represent new alternative isoforms of known genes. First, we checked our ability to detect known splices in our data (Additional file 1: Table S5). We found that the majority of known splices can be detected. Pooling all the experiments together, we detected ~75% of about 2x10^5^ known splices. The sensitivity of splicing detection decreases for individual samples, but we still observed at least 50% of known splices in each of our individual samples.

We then considered all annotated exons within each gene and constructed a list of all possible splice junctions (~2 × 10^6^ junctions; see Materials and Methods). Overall, we found support for about 13,000 new junctions (Additional file 1: Table S6 and Additional file 5). Although many of these novel splicing variants are expressed at a low level, some were rather abundant.

**Figure 5:**
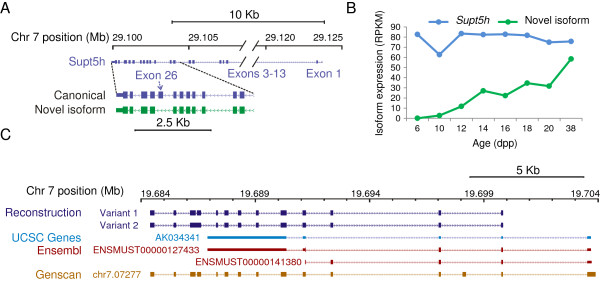
**Gene and isoform predictions.** (Top) Novel alternative splicing of transcription elongation factor *Supt5h* differentially expressed in spermatogenesis. We see an ORF-preserving exon skipping event **(A)** that is frequently detected in samples after 12 dpp **(B)**. **(C)** Predicted transcripts (variant 1 and variant 2 differ only by one retained intron). This prediction is based on our RNA-Seq data in combination with UCSC and Ensembl annotations and Genscan computational gene predictions. Only the protein coding part of the predicted transcripts is shown. The respective lengths of the predicted proteins are 566aa and 589aa. Both of these variants have a HMG box, which is a DNA-binding domain, towards their C-terminus, which is completely missed by the UCSC and Ensembl gene models. Expression at this locus peaks sharply at 12dpp. Note that there are a few differences in the boundaries of some exons between the predicted transcripts and the known annotation, which are invisible here.

We found that 38% (4,619 out of 12,272) of the novel intra-isoform exon skips (splice junctions between non-neighbor exons, per known isoform annotation) preserve an open reading frame (ORF). The extent of ORF preservation is higher for highly-expressed novel splices, with five out of the top six splices – in genes *R3hdm1* (single-stranded nucleic acid-binding), *Atp8b3* (ATPase), *Usp34* (ubiquitin hydrolase), *Wt1* (transcription factor essential in development of urogenital system) and *Supt5h* (see below) – being ORF-preserving. Importantly, these abundant novel ORF-preserving splices constitute a significant fraction of the total gene expression. On average, the maximal proportion of novel isoform expression is above 43%, for splice junctions with at least 10 independent reads. One of the particularly abundant novel variants is the skipping of a coding exon (chr7:29,101,301-29,101,460) in the *Supt5h* gene with the preservation of the ORF (Figure 5). This gene participates in the regulation of mRNA processing and transcription elongation by RNA polymerase II. Expression of this novel splice junction at 14 dpp to 38 dpp is on the order of 20 to 60 RPKM, rising at later time points, while *Supt5h* expression as a whole is between 60 to 85 RPKM for 6-38 dpp. Hence, this novel splice isoform can contribute a significant fraction to the total gene’s expression, especially later in spermatogenesis. This suggests that this *Supt5h* isoform might be important for sperm maturation.

In the novel splices, we asked how many exons are skipped, when compared to the known isoforms. Typically 85 to 90% of our novel splices involve skipping one exon (as reported for other tissues), and about 6-8% skip two exons (Additional file 1: Figure S6). While most novel splices skip one exon, there are exceptions like the skipping of exons 5 through 15 in the spermatogenesis-associated gene Spata5. However, the ORF is not preserved. Finally, we detected 740 novel exon-exon junctions that could only be formed by splicing exons present exclusively in alternative known isoforms of a given gene. Out of these 740 junctions, 79 (11%) preserve the ORF, suggesting that they represent functional transcript variants.

### Gene predictions

Since RNA-seq data are not restricted to annotated genes, we looked at regions of high expression outside of any of the UCSC knownGene, refGene and xenoRefGene lists. Interestingly, for many such regions with a high level of expression there was an associated (overlapping) Genscan gene prediction. Hence we asked whether we can detect meiotic expression of computationally predicted putative genes. We compared RNA-Seq reads to those expected from gene models of the Ensembl and Genscan databases. Our data yielded support for 70 gene models from Ensembl, that don’t overlap with annotated genes found in the UCSC knownGene, refGene and xenoRefGene (non-mouse genes) tables. To ensure a high level of specificity, we demanded that we observe at least one predicted splice site for the models considered which would constitute an additional indication of transcription and transcript processing (Additional file 6). Similarly, we found support for 97 Genscan gene models that do not overlap with any known genes (Additional file 7). Most of these models are expressed at low levels, with only 41 showing maximal expression above 2 RPKM (in comparison, the average expression level of known genes is around 10 RPKM, and the uniform genome coverage would result in ~0.3 RPKM). One interesting example is the Genscan model *chrX.595* demonstrating a meiotic upregulation, peaking at 12 dpp with 120 RPKM. This model has 16 exons; we see 11 splice junctions, 5 of which are splices between neighboring exons. Therefore, there is evidence for alternative splicing as well. We note that out of the 97 Genscan gene models, only 8 overlap the 70 Ensembl models described above.

Recently, a class of large intervening non-coding RNAs (lincRNAs) has been described and studied. They are proposed to form ribonucleic-protein complexes acting in numerous cellular pathways. In mouse, these lincRNAs have been obtained from four cell types not involved in spermatogenesis [37-39]. We asked whether we had evidence for expression of any of these lincRNAs in our data, and how it changed over time. Combining lincRNA genomic regions and exonic structures reported in [37,38] yielded 6,129 gene structures that don’t overlap known UCSC genes. Given that these gene structures were not manually curated, we conservatively restricted our analysis to long structures with high and variable expression (total exon length > 1000 bases, RPKM > 5, and at least a 5-fold change in expression), in order to focus on potentially more reliable variants. This filtering yielded 59 structures (many partially overlapping), which can be interesting candidates for future studies (Additional file 8).

Since lincRNAs are defined as non-coding transcripts, we also analyzed known UCSC genes that are annotated as non-coding. There are 6,544 such genes. 1,231 of them have maximal RPKM > 2, at least a 2-fold change in expression and an mRNA length at least 100 bases; 254 of these transcripts overlap lincRNAs. With stricter thresholds, 228 of the 6,544 non-coding genes have maximal RPKM > 5, fold change > 5 and an mRNA length at least 1 kb; 50 of them overlap lincRNAs. As we are mostly interested in meiosis, gene *AK034341* caught our attention, as its expression peaks quite sharply at 12 dpp with around 30 RPKM. We noticed that this gene has two isoforms in the Ensembl and Vega annotations, both with coding potential. Moreover, our RNA-Seq data suggested that none of these annotations accounted for the observed coverage toward the 3′ end of the transcript. Computationally predicted Genscan transcript *chr7.07277* was closer to the observed RNA-Seq read coverage. We therefore performed a detailed reconstruction of this transcript. The result is mostly the merger of the 5′ part from the Ensembl/Vega annotations with the 3′ part from the Genscan predictions, with additional corrections to exon boundaries in some exons. The resulting two possible very similar transcripts are protein coding, and possess an HMG-box, a DNA-binding domain, towards their 3′ end (Figure 5). Their predicted protein lengths are 566 aa and 589 aa (Additional file 9). Given that its expression profile is highly similar to that of the *Prdm9* gene, this novel gene is an interesting candidate for further study.

### Polyadenylation

**Figure 6:**
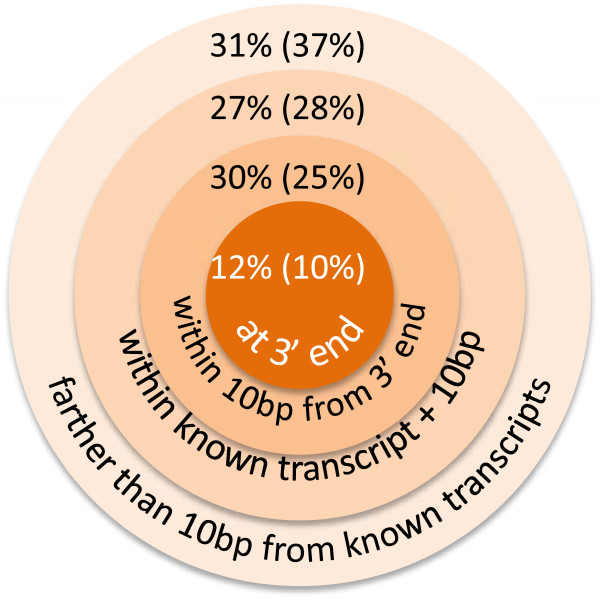
**Detection of polyadenylation.** The proportion of the polyadenylation sites perfectly matching the annotated 3′ ends of known genes is shown in the center of the diagram. Subsequent rings going outwards show the percentage of tentative polyadenylation sites successively further away from the known 3′ ends. The percentages without brackets are based on the total of 5,229 sites found with zero mismatches, while bracketed percentage values correspond to the total of 6,801 sites allowing up to one mismatch – see text for details.

One of the mechanisms regulating gene expression is alternative polyadenylation [40]. While our experiments were not specifically designed to address this question, our data contain multiple reads covering polyadenylation sites. Hence, we asked whether we could detect alternative polyadenylation during spermatogenesis and to what extent. We implemented a mapping strategy based on partial mapping of previously unmapped reads, together with observation of a polyadenylation tail and a polyadenylation signal (see Materials and Methods). Mapping reads to the whole genome, we identify 5,229 candidate polyadenylation sites (if we demand zero mismatches; if we allow for ≤1 mismatch, the corresponding number is 6,801 – see Figure 6 and Materials and Methods). 3,623 (4,269) candidate sites lie within known gene transcripts ±10 bases, and of those 2,222 (2,365) are at 10 bases or less from the closest known 3′ transcription end. 638 (676) candidate sites are right at the known 3′ transcript end (Figure 6). There are over 20,000 genes, many of which have multiple isoforms/transcripts, some of which have distinct 3′ ends. As there are 49,409 known transcripts we detect only ~10% or less of known 3′ ends.

In summary, out of 5,229 candidate sites, 2,222 are within 10 bases from known sites, while 3,007 sites are farther away. About half of the latter are within known gene bodies. Such sites are potentially novel sites of alternative polyadenylation. Extrapolating these numbers to all genes, we estimate that there can be over 30,000 alternative polyadenylation sites active through spermatogenesis. This finding is consistent with recent work highlighting the abundance of alternative polyadenylation [40-42].

### Meiotic sex chromosome inactivation

**Figure 7:**
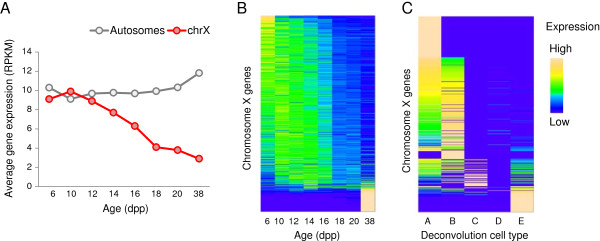
**The X chromosome is transcriptionally silenced in spermatogenesis as a result of Meiotic Sex Chromosome Inactivation. (A)** The average expression of X-linked genes decreases after 12dpp, and eventually drops by a factor of ~3 in adult testes. **(B)**, **(C)** Heatmaps of X-linked genes. Only genes selected for temporal clustering are shown **(B)**. The drop in gene expression, relative to genome-wide expression, is evident starting at around 16dpp, the pachytene stage. In the adult testes, at 38dpp, many genes that have not been active during the earlier stages of the first wave of spermatogenesis are turned on. This is also evident from the deconvolution calculations **(C)**.

Gene expression on the X chromosome is strongly repressed in pachytene via a process known as meiotic sex chromosome inactivation (MSCI) [43-45]. Our data show clear evidence of MSCI – gene expression of X-linked genes drops dramatically beginning around 16 dpp (Figure 7) and there are no X-linked genes in the late meiotic clusters 6 and 7 (Additional file 1: Figure S7). Similarly, our deconvolution results show only 15 X-linked genes with pachytene (D) expression, and in all these cases, except one (*Gm15070*, which is a hypothetical protein), the estimated expression in D is small compared to the expression in some of the other cell types. Post-meiotically, however, the X chromosome is no longer strongly repressed and many X-linked genes belong to temporal cluster 8 and are expressed in cell type E.

### RNA Polymerase II ChIP-Seq

**Figure 8:**
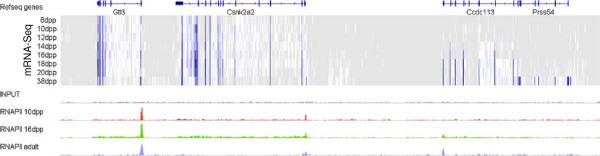
**Example of mRNA-Seq and Pol II ChIP-Seq coverage in a 164 kb genomic window on chromosome 8 (chr8:97,939,000-98,103,500).** mRNA expression is high in blue and absent in grey. There is Pol II accumulation at promoters of genes, and an elevated Pol II signal can be seen along gene bodies as well. Here, some genes are expressed throughout the whole time course, while others are initially virtually silent. Consistent with these expression profiles, the presence of Pol II at corresponding promoters occurs at different time points.

RNA polymerase II is responsible for transcription of pre-mRNAs as well as various non-coding RNAs. To study the correlation of Pol II binding with gene expression in spermatogenesis, we performed two Pol II ChIP-Seq experiments, on testes of 10 and 16dpp mice, and used a third, published dataset for adult testis [46]. Typically, we see a clear Pol II binding signal at ~36% of annotated transcription start sites (TSS’s), and sometimes an increased signal along genes (Figure 8). There is a noticeable correlation between the level of gene expression and the strength of the Pol II signals measured at 10 and 16 dpp – Spearman correlation is in the range 0.45 – 0.8, and is highest at or around the matching time point. Significantly, the correlation is high not only for a Pol II signal around gene TSS’s, but also in the gene bodies, 1 kb and farther downstream from TSS’s (Additional file 1: Figure S8).

We detected accumulation of Pol II at ~10,000 canonical transcription start sites (TSS’s) and ~600 genes use known alternative TSS’s instead. Of the remaining genes, 100-200 have strong Pol II peaks within the gene body. Overall, ~1,000 strong Pol II peaks lie within gene bodies (more than 1 kb away from TSS), and there is a similar number of strong peaks farther than 1 kb from annotated gene bodies. Using more comprehensive sets of predicted Pol II promoters and TSS locations ([47,48] and a table of mRNAs from the UCSC browser (all_mrna)), we observed an increased overlap with our Pol II signals (Additional file 1: Table S7). We also observed Pol II in CpG islands and overlapping histone 3 lysine 4 trimethylation (H3K4me3) marks [49], associated with active promoters (Additional file 1: Table S7).

We found that the majority of active gene promoters (TSS’s) have an accumulation of Pol II at all three time points (10 and 16 dpp, and adult) (Additional file 1: Figure S9). About half of the 19,858 genes with unambiguously assigned TSS’s have at least one of their possibly multiple TSS’s occupied by Pol II at at least one time point. Dissecting the genes by their expression cluster, we saw the expected picture of a higher 10dpp Pol II promoter occupancy in early expression clusters and a higher adult Pol II promoter occupancy in late expression clusters (Additional file 1: Figure S10). We also considered genes with roughly constant expression (maximal RPKM above 2 and fold change less than 2, denoted as ‘const’ cluster), and genes with low expression (maximal RPKM below 2 in all the samples, denoted as ‘low’ cluster). 88% of genes with roughly constant expression (‘const’ cluster) have their promoters occupied in at least one of the three time points considered, and 82% in all three time points. In contrast, only 7% of weakly or non-expressed genes (‘low’ cluster) have Pol II signals at their promoters at some point, and these signals are weak.

To address whether there is promoter occupancy by Pol II but no or low expression, we focused on late expression clusters 7 and 8, and looked for genes with a Pol II signal at 10dpp. This yielded 106 out of 1179 genes for cluster 8 and 230 out of 711 genes for cluster 7. We also considered genes from cluster 8 with a Pol II signal at 16dpp – 154 out of 1179. Using DAVID [50,51], we found that these lists of genes are enriched in annotations like cytoskeleton, intracellular non-membrane-bounded organelle, microtubule, cell projection, cilium and related terms, with annotation clusters enrichment scores between 1.53 and 4.39. We note that these annotation terms are enriched even when the whole corresponding expression cluster is used as a background (instead of the whole set of mouse genes). Thus it seems likely that genes responsible for sperm motility have significant accumulation of Pol II around their TSS’s early on, but are expressed at relatively low levels until later. This observation is similar to the situation of a poised Pol II, which is quite ubiquitous during development [52,53].

We also analyzed cases of gene expression without clear Pol II at annotated TSS’s. We considered expression clusters ‘const’ and 8 (Additional file 1: Figure S10) as one would expect to see Pol II at TSS’s of all ‘const’ genes, and cluster 8 has the least percentage of genes with Pol II at TSS’s. For the 'const' expression cluster most of the genes in question have in fact nearby Pol II at one of their annotated TSS’s, which have been omitted due to either the ambiguity of assigning a TSS interval to a gene (e.g., in cases of bidirectional promoters), or because short gene transcripts were excluded; in some cases, the gene expression, or the mappability of the TSS region is low; in yet other cases there seems to be an incorrect annotation, for example defining part of one transcript as a separate transcript with its own TSS, leading to disagreements between the RefSeq and UCSC annotations. For expression cluster 8, in addition to the above, there are cases of overlapping transcripts on opposite strands; sometimes Pol II signals are too weak to be called (genes with statistically significant Pol II signal near their TSS’s have a much higher maximal RPKM: median 51 vs. 10 for those without such signal). Another factor is that the adult Pol II ChIP data [46] used by us seems to correlate better with gene expression at 18 and 20dpp than with 38dpp. Certainly, some genes use un-annotated alternative TSS’s, either within or outside of the annotated gene transcripts. One possible example is the *Tex16* gene whose putative un-annotated TSS lies ~200 kb upstream of its annotated 5′ end (Additional file 1: Figure S11).

## Discussion

### Identification of meiotically expressed genes

To define genes expressed in meiosis we sequenced the transcriptome of murine testes at eight time points during the first wave of spermatogenesis. We then applied two approaches for the identification of genes expressed during meiosis: clustering of temporal gene-expression profiles and computational deconvolution of temporal expression patterns into cell-type specific expression profiles. In four out of eight temporal expression clusters median expression rose between 10 and 20 dpp - a time period that corresponds to the meiotic stages of spermatogenesis. We thus designated genes from these clusters as meiotically expressed. A comparison of this gene list with previous publications allowed us to define 1,048 novel meiotic genes which were not previously analyzed by microarray studies (Additional file 3). As expected, our novel meiotic genes are significantly enriched in such meiosis-related Gene Ontology categories as microtubule-based movement and response to DNA damage stimulus, among others (Additional file 1: Figure S12).

Our experimental measurements of gene expression levels constitute bulk expression estimates from heterogeneous samples containing various proportions of germ and somatic cells. To estimate gene expression levels in individual cell types we developed a computational deconvolution approach (Figure 3). Using this deconvolution procedure, we found 375 genes (262 protein-coding) that are exclusively expressed in meiotic cell fractions C and D. Out of them 205 (111 protein-coding) are novel. When we compare our temporal clustering classification with the deconvolution predictions, we find that 920 out of 1,048 novel meiotic genes identified via clustering of the temporal gene expression profiles are highly expressed in the cell types that contain meiotic cells, B, C and D (with 567 in C and D). Thus, both of our analytic approaches define a consistent set of novel meiotic genes.

Although the vast majority of annotated gene models in mouse have experimental support, there are numerous gene models predicted only using computer tools. Here we find direct experimental support for 159 computationally predicted transcripts that don’t overlap known genes. Also, based on partially correct computational models in the locus corresponding to the known non-coding gene *AK034341,* we predict a protein-coding putative gene of 566/589 aa with a DNA binding HMG box domain (Figure 5). The expression profile of this gene shows a sharp peak of transcription in meiosis, between 10 and 14 dpp.

### Meiotic sex chromosome inactivation

It is clear that MSCI suppresses X-linked gene expression in male meiosis (see above and [34,45,54]). Previously it has been suggested that MSCI persists beyond meiosis into spermiogenesis [34]. Our temporal profiling results and *in silico* deconvolution allows us to estimate post-meiotic gene expression of X-linked genes and test the hypothesis that the X chromosome is still inactivated past meiosis. It has been reported [34,54] that 13% to 18% of X-linked genes are expressed after the completion of meiosis in mouse. In our analysis, only 27% of X-linked genes are expressed in the post-meiotic deconvolution cell type E, compared to 46-59% of autosomal genes. In contrast, 85% of X-linked and 84-88% autosomal genes are expressed in the mostly somatic cell type A. Moreover, 12.4% of X-linked genes are expressed mostly in the post-meiotic deconvolution cell type E (Figure 4), a proportion that is lower than that for autosomal genes (13.3-17.5%). Thus, even though MSCI is relaxed after the completion of meiosis as suggested before [34], the X chromosome is transcriptionally suppressed in post-meiotic cells. This is consistent with recent work [13].

### Dynamics of RNA polymerase II binding patterns and their relation to gene expression

We supplemented our RNA-Seq data with RNA polymerase II ChIP-Seq. While most promoters of expressed genes have a discernible Pol II ChIP-Seq signal, thousands of locations far from annotated gene promoters display accumulation of Pol II. These locations can mark novel promoters or enhancers, or clusters of small RNAs. Indeed, comparison of the Pol II signals at 10dpp and 16dpp clearly identified activation of pachytene piRNAs after 10dpp (Additional file 1: Figure S13). Consistent with previous findings [55-57], we found an enrichment of Pol II in our 16dpp (pachytene) sample in 80 out of 94 piRNA clusters.

Previous studies have shown that in development, gene promoters often harbor a poised Pol II at genes that remain suppressed until a certain developmental stage [52,53]. Because spermatogenesis is also a developmental process, we hypothesized that a similar phenomenon might be happening here as well. While our experimental procedures (ChIP-Seq) cannot determine if the observed Pol II is transcriptionally engaged [53], we find that genes likely to be associated with sperm motility have a Pol II signal around their TSS’s as early as at 10dpp. This suggests that promoter poising is common in spermatogenesis.

## Conclusions

Here we created a comprehensive reference dataset of gene expression in mouse spermatogenesis. We analyzed expression in males aged from 8 dpp to 38 dpp effectively covering all stages of spermatogenesis from pre-meiotic cells to spermatozoa. We then computationally detected genes expressed pre-meiotically, early and late in meiosis and in sperm. Since previous studies of gene expression during spermatogenesis in mouse were primarily performed using microarrays, only a subset of the genes was analyzed. Unlike microarrays, our data cover essentially all mouse genes. An additional advantage of RNA-Seq compared to microarrays is superior sensitivity. These two factors allowed us to define over a 1,000 novel meiotically-expressed genes. Furthermore, our data show that alternatively spliced isoforms are abundantly represented through spermatogenesis. In total, we found more than 13,000 of novel alternative splices some of which are highly expressed in meiosis. To further enhance our dataset and better define promoters we supplemented transcription profiling with RNA polymerase II ChIP-Seq at three time points. This allowed us to describe temporal dynamics of Pol II behavior in spermatogenesis and detect numerous regions enriched for Pol II away from annotated genes. Taken together, we believe that our integrated dataset is a valuable resource for further studies of gametogenesis and meiosis in vertebrates.

## Methods

### Sample preparation

For mRNA-Seq, whole testes mRNA extracts from murine pups [58] aged 6, 10, 12, 14, 16, 18, 20 and 38 days post partum (dpp) were obtained. Total RNA was purified using a Total RNA Isolation Mini Kit (Agilent) according to the manufacturer’s instructions. 20 μg of total RNA was loaded on 100 μl oligo(dT) Dynabeads (Dynal) and polyA + fraction was eluted in 15 μl of DEPC-treated water. 0.5 μg aliquots of polyA + RNA were fragmented in total volume of 10 μl of RNA fragmentation buffer (40 mM Tris-Acetate, pH 8.1, 100 mM KOAc, 30 mM MgOA) for 2 min at 95°C. Samples were diluted to 100 μl using DEPC treated H_2_O and immediately subjected to ultrafiltration on Amicon YM-30 filters. First and second strand cDNA synthesis was performed using a SuperScript double-stranded cDNA synthesis kit (Invitrogen) using 150 ng of fragmented RNA and 500 ng of random hexamers (Promega) as recommended by the manufacturer. The standard Illumina protocol was used to construct the sequencing library.

RNA polymerase II (Pol II) ChIP procedure follows the standard protocol from manufacturer. Whole testes extracts were collected from 10 and 16 dpp pups, and immediately cross-linked with 1% paraformaldehyde in 1 × PBS for 10 minutes at room temperature. Cross-linked cells were washed with pre-lysis buffer A (0.25% Triton X-100, 10 mM EDTA, 0.5 mM EGTA, 10 mM Tris-HCl pH 8) and pre-lysis buffer B (0.2 M NaCl, 1 mM EDTA, 0.5 mM EGTA, 10 mM Tris-HCl pH 8). Cells were pelleted and sonicated in SDS lysis buffer (1% SDS, 10 mM EDTA, 50 mM Tris-HCl pH 8 and 1x Protease Inhibitor Cocktail (Roche) for 16 cycles at 15 sec on and 15 sec off in a sonicator water bath (Bioruptor, Diagenode) at 4°C. After that fragmented chromatin samples were dialyzed with ChIP dilution buffer (0.01% SDS, 1.1% Triton X-100, 1.2 mM EDTA, 16.7 mM Tris-HCl, 167 mM NaCl), samples were incubated with antibody-coated magnetic beads (Dynabeads M-280, Invitrogen) overnight at 4°C. The antibodies used were rabbit polyclonal anti-RNA polymerase II CTD repeat YSPTSPS (phospho S5) (Abcam ab5131) for the 16 dpp sample, and rabbit polyclonal anti-RNA polymerase II (N-20) (Santa Cruz sc-899) for the 10 dpp sample. Magnetic beads were washed for 5 min once each in low-salt immune complex wash buffer (0.1% SDS, Triton X-100, 2 mM EDTA, 20 mM Tris-HCl pH 8, 150 mM NaCl), high-salt immune complex wash buffer (0.1% SDS, Triton X-100, 2 mM EDTA, 20 mM Tris-HCl pH 8, 500 mM NaCl), LiCl immune complex wash buffer (250 mM LiCl, 1% NP-40, 1% deoxycholic acid (sodium salt), 1 mM EDTA, 10 mM Tris pH 8.0) and then twice in 1x TE for 5 min at 4°C. Immunoprecipitated chromatin samples were eluted in elution buffer (1% SDS, 100 mM NaHCO_3_) for 15 min at 65°C and reverse cross-linked overnight at 65°C with 200 mM NaCl. Proteinase K treated reverse cross-linked DNA was purified with a MinElute PCR purification kit (Qiagen) and then the DNA concentration was measured using a Qubit dsDNA HS assay kit (Invitrogen).

### Data acquisition

The sequencing was done on an Illumina Genome Analyzer. The summary of reads for mRNA-Seq is given in Additional file 1: Table S1. We acquired 36 bases long, single end reads. The first and the last two bases in each mRNA-Seq read were discarded, as having a high error rate relative to the rest. The first two bases are imprecise due to random primer mismatches, while the last two are due to the limitations in the instrument and reagents used, leading to higher error rates. Similar procedures were used to align Pol II ChIP-seq data, although without discarding the first and last bases. The data is available through the NCBI’s Gene Expression Omnibus using the GEO Series accession number GSE44346.

### Data analysis

All annotations were downloaded from the UCSC genome browser database. Specifically, we used the Mus musculus mm9 genome version, with gene annotation version 4 tables for the gene model definitions and for the selection of one isoform per gene, respectively. Other tables were used as well, as described in the text.

Reads were aligned using ELAND short read alignment software for the Illumina Genome Analyzer (version 1 was used for mRNA-Seq reads and version 2 for Pol II ChIP-Seq). Only the uniquely aligned reads passing the quality filtering were selected for calculations reported here. Post-alignment analysis was done using custom scripts written in Perl and R languages, in conjunction with local MySQL database server. We calculated expression levels of all known genes for all 8 samples using the RPKM (Reads Per Kilobase per Million of reads) measure of gene expression [59].

For Pol II ChIP-Seq, we used three data sets, at 10 dpp, at 16dpp and at 8 weeks (adult mice). The adult set was downloaded from GEO, accession numbers GSE36027 and GSE29184 [46]. Pol II ChIP-Seq data was analyzed using MACS (version 2.0.9) [60], bedtools (version 2.15.0) [61], CCAT (version 3.0) [62], and various custom scripts in Perl and R. To calculate the correlation of gene expression with Pol II signals around gene TSS’s and along gene bodies, background-corrected read counts within 250 bp of annotated TSS’s, and along gene transcript excluding 5′-most 1 kb were considered, respectively. Short (<1 kb) gene transcripts were discarded. The correction for a non-specific signal (background or noise) was done by estimation of the noise fraction with CCAT, as well as by comparison of ChIP and input signals in regions distant from annotated genes, and also by maximization of correlation by varying the noise fraction. All approaches produced similar estimates. Given the noise fraction, the specific signal at a locus was estimated as the raw ChIP signal from which the estimated non-specific part is subtracted, given that there is a statistically significant difference between raw signal and input (binomial test, false discovery rate 0.05). If the significance threshold was not reached the specific signal was set to zero.

For the presence of Pol II peaks near TSS’s, we checked for overlap of MACS-derived peaks with a TSS interval file containing all annotated TSS-centered 500 bp intervals with overlapping intervals merged, having excluded TSS’s derived from short gene transcripts (unprocessed transcript length ≤1 kb). This yields 27,775 TSS-proximal intervals. 19,858 genes could be unambiguously assigned to these intervals; the ambiguous cases with divergent transcription were excluded. Although the total number of Pol II peaks found in the three samples (10 dpp – 20,882, 16 dpp – 68,347 and adult – 15,790), varies significantly, and the antibodies used are different, the fact that there are between 9,000 to 11,000 TSS-proximal peaks in all these samples serves as the basis for their use and comparison.

#### Clustering

To cluster the temporal gene expression profiles we used the k-means algorithm as well as the HOPACH algorithm [63], as they are implemented in the R statistical software (http://www.R-project.org). There are at least a few issues to consider in clustering. One is how to normalize gene expression. In this paper, each gene expression is normalized so that the sum of squares for all points equals 1. The advantage is that zero expression remains zero. Together with the Euclidean distance, this approach is basically equivalent to using the “cosangle” distance measure between the original gene expression profiles of two genes [63,64].

Another issue to consider is how many clusters to choose. This of course depends on how detailed we want the clustering to be. Application of HOPACH algorithm [63] results in 8 clusters for the first level of this hierarchical clustering approach. Going further down identifies more than 400 “main clusters”. Our purpose here is to get a genome-wide view of the gene expression, and therefore we limited the number of clusters to single digits. Using k-means with 8 clusters and the same distance measure yields very similar results and we use this clustering in the paper.

We also used k-means with a cosangle distance measure to cluster gene expression reported in [8] and [13], as discussed in the text.

#### Deconvolution

In the deconvolution procedure we assume that gene expression of most of the genes in a given cell type is cell-autonomous, and does not change much with the age of a mouse. If this were not true we could in principle argue that we are dealing not with one but with many cell types, each having its characteristic gene expression profile. The question is therefore reduced to the determination of the number of distinct cell types.

Here, as for the gene expression calculations, we pick only one isoform per gene. Utilizing the digital nature of our sequencing, we select genes that have at least 100 reads (hits) within the canonical mRNA transcript, as an accuracy cutoff. This yields 14259 genes for the deconvolution analysis. Taking the number of reads into account allows us to estimate the standard error in this number, which we assume to be its square root, following the Poisson model. Hence, we can develop a weighted least squares algorithm as described below.

Let *N*_*ijk*_ be the number of hits from sample *i* (out of our 8 temporal samples) to gene *j* that came from cell type *k. N*_*i*_ = ∑ _*j*,*k*_*N*_*ijk*_ is the number of hits from sample *i*, and *N*_*ij*_ = ∑ _*k*_*N*_*ijk*_ is the number of hits from sample *i* to gene *j*. Let *n*_*ijk*_ ≈ *N*_*ijk*_/*N*_*i*_ denote the fraction of hits from gene *j* and cell type *k* in sample *i*. It can be expressed as *n*_*ijk*_ = *w*_*ik*_*n*_*kj*_, where *w*_*ik*_ ≈ (∑_*j*_*N*_*ijk*_)/*N*_*i*_ is the fraction of hits from cell type *k* in sample *i* and *n*_*kj*_ ≈ (∑_*i*_*N*_*ijk*_)/(∑_*i*,*j*_*N*_*ijk*_) is the fraction of hits in cell type *k* coming from gene *j*. Note that independent estimates of this fraction could be imagined, one for each sample *i*, if we do not sum over *i*; these would vary from sample to sample due to fluctuations. In the formulas above we used approximate equalities to reflect the fact that on the right-hand side are the data-based estimates of these quantities.

We also assume that there are indeed *K* cell types, that gene expression within each cell type is identical in all samples irrespective of the tissue composition, and that there are more samples than cell types (otherwise the error terms cannot be estimated), or that these assumptions are good approximations. We thus obtain $N_{\mathrm{ij}}=\sum_{k}^{K}N_{i}w_{\mathrm{ik}}n_{\mathrm{kj}}+\sqrt{N_{\mathrm{ij}}}\epsilon_{\mathrm{ij}}$ where *K* is the number of cell types assumed, and all “normalized” errors *ϵ*_*ij*_ are assumed to have mean zero and variance 1. Dividing the last equation by *N*_*i*_ and transcript length *L*_*j*_, measured in bases, yields $S_{\mathrm{ij}}=\sum_{k}^{K}w_{\mathrm{ik}}C_{\mathrm{kj}}+\left(\frac{\sqrt{N_{\mathrm{ij}}}}{N_{i}L_{j}}\cdot 10^{9}\right)\epsilon_{\mathrm{ij}}$, where $S_{\mathrm{ij}}=\frac{N_{\mathrm{ij}}}{N_{i}L_{j}}\cdot 10^{9}$ is the RPKM of gene *j* in sample *i*, and $C_{\mathrm{kj}}=\frac{n_{\mathrm{kj}}}{L_{j}}\cdot 10^{9}$ is the RPKM of gene *j* in cell type *k* – exactly what we are looking for.

The normalized error terms are $\epsilon_{\mathrm{ij}}=\frac{N_{i}L_{j}}{10^{9}\sqrt{N_{\mathrm{ij}}}}\left[S_{\mathrm{ij}}-\sum_{k}w_{\mathrm{ik}}C_{\mathrm{kj}}\right]$ and we want to minimize $\sum \epsilon_{\mathrm{ij}}^{2}$ (when *N*_*ij*_ = 0 in the last formula it is replaced by 1). For given cell type contributions *w*_*ik*_ we take the last sum over *i*, for any gene *j* separately. This will yield the estimates for *C*_*kj*_. Given these estimates, we can update the *w*_*ik*_ by summing the $\epsilon_{\mathrm{ij}}^{2}$ over *j*, for each sample *i* separately. This procedure of optimizing for *C*_*kj*_ and then for *w*_*ik*_ can be run iteratively. If the algorithm works, we should expect the normalized error terms to have typical values of the order of 1. With our data, most noticeable changes occur in first two to three iterations, after which the results stabilize.

There are constraints to the allowed values for both for *C*_*kj*_ and *w*_*ik*_: they must be non-negative. Negative values are replaced by zero. More precisely, the corresponding predictor with the most negative coefficient is excluded from the list of predictors, and then the minimization is performed again with the number of predictors reduced by one. If the significance p-value for a given predictor is above 0.05 it is also excluded, and minimization is repeated. The minimization procedure is stopped if either all the remaining predictors have positive coefficients with p-values below 0.05 or there is only one predictor left (in this case its coefficient is always positive). In addition, ∑ _*k*_*w*_*ik*_ =1 as we assume we include all the cell types present. This normalization is enforced after each iteration (for the reported deconvolution with 5 cell types, we also restrict the cell type fractions at 6dpp so that contribution from B is below 4%, while C, D and E are zero; this leads to only minor differences with the unrestricted analysis). The distribution of p-values for remaining predictors is shown in Additional file 1: Figure S14.

We are fully aware that due to the stochastic nature of our algorithm, namely, due to data-dependent elimination of some predictors, the standard p-value calculations are incorrect. Still, we use them formally as we are not familiar with a sensible alternative suitable for our case, except bootstrapping, as discussed below. Additionally, our goal here is not to test hypotheses, but to find for each gene the cell types that would explain its expression profile sufficiently well. We believe that, perhaps with some exceptions, our goal is achievable via the described procedure.

A question that arises from the deconvolution analysis is the reliability of the predicted cell type-specific gene expression. We compare our predictions to microarray measurements from a cell-sorted study [6], and find them in reasonable agreement (Additional file 1: Figure S15). We also find a reasonable agreement between our deconvolution predictions and a recent study using RNA-seq to measure gene expression in cell-sorted samples [13] (Additional file 1: Figure S3). We note that on a genome-wide scale, it is doubtful that any approach, including direct cell type-specific expression measurements, will yield a completely unambiguous classification.

To study the stability of our algorithm with respect to perturbations to gene expression, we performed a bootstrap. Assuming a Poisson distribution of hits for a given gene, with unknown rate, and using a Bayesian uniform rate prior yields a negative binomial distribution of hits with a mean n + 1 and a variance 2(n + 1), where n is the measured number of hits (to have a mean n and a variance 2n, the prior should be inversely proportional to rate; however, in that case, if n = 0 the simulated distribution will always produce 0 as well). For 102 bootstrap runs, starting with initial cell type fractions and with perturbed gene expressions and running for ten iterations, we calculated the presence fractions for each gene, which are the fractions of bootstrap runs in which this gene is found expressed, for each cell type considered. We found that for each cell type there is a well-defined bimodal distribution of presence fractions, with maxima near 0 and 1 (i.e., genes are either consistently predicted not expressed, or expressed, in a given cell type). We define a consensus prediction by a threshold of 0.5: if the presence fraction is greater than 0.5 the gene is present, and vice versa. The difference between the original (non-perturbed) and consensus results is small: the ratio of genes with different status to those with the same status (either expressed or not) is below 6% for all five cell types. If we define genes with a presence fraction of 95% or more as confidently expressed, and genes with a presence fraction of 5% or less as confidently silent, then the ratios of confidently silent to consensus silent are between 0.61 and 0.73 for the five cell types, and the ratios of confidently expressed to consensus expressed are 0.92, 0.58, 0.48, 0.71 and 0.73 for A, B, C, D and E, respectively. These results show that for most of genes the algorithm produces stable predictions.

Based on the estimated cell type-specific expression, and on the cell type fractions, it is possible to reconstruct the temporal expression patterns of the genes. We compare such reconstructed expression patterns after 1 and 10 iterations with the original, observed expression (Additional file 1: Figure S16 and S17). The agreement is excellent in both cases, and is better after 10 iterations, especially for 38dpp. While such an agreement is not by itself a full guarantee of the validity of our approach, it is necessary condition for it.

Another useful way to validate our cell type-specific gene expression predictions is to see how stable the predictions are between different iterations, and between different numbers of the cell types considered. We consider 5, 6 and 7 cell type calculations, at iterations 1, 3 and 10 and at different p-value cutoffs of 0.05 and 0.001. For 5 cell types, Bs (types A and B spermatogonia) and Bl (pre-leptotene and leptotene spermatocytes) have been combined into one type, B (cell fraction profiles for Bs and Bl are quite similar; a similarity for the profiles was one of the guides to establish the initial cell type groups for our analysis, based on [25,32]). For 7 cell types, A has been split into As and Ap, which are Sertoli and primitive spermatogonia A, based on [25,32].

We find that 5- and 6-cell type calculations agree well (Additional file 1: Table S8) – for any given iteration there are typically over 11,000 genes (out of 14,259; >77%) with a compatible predicted expression (compatibility/consistency means that if a gene is found expressed (silent) in a certain cell type in one calculation, it has to be expressed (silent) in the corresponding cell type(s) in the other calculations considered in the comparison as well). When, in addition, we look at compatible expression for different iterations (3 and 10) we still find over 50% in agreement. The comparison of 6- and 7-type is less consistent, indicating that 7 cell types are too many for the data (Additional file 1: Table S8).

Additionally, we calculate Pearson and Spearman correlation coefficients for different cell type-specific gene expression vectors (with elements being individual genes) between iterations, for 5- and 6-cell type calculations (Additional file 1: Table S9). The Pearson correlation is dominated by highly expressed genes, while the Spearman correlation results are close to the Pearson correlation for normalized gene expression (when sum of squares of expression RPKM over the cell types is normalized to 1 for each gene; note that only genes with non-zero expression in at least one of the samples were selected for deconvolution, so there is no ambiguity with non-expressed genes). The Pearson correlation is above 0.5 for both 5 and 6 cell types, while the difficulty in distinguishing Bs and Bl types is reflected in the low value of the Spearman coefficient especially for Bs in the 6-type calculation. 5- vs. 6-cell type cross-table correlations demonstrate similar tendencies. Overall, we find these results satisfactorily provide confidence in our deconvolution algorithm predictions.

We note that in the absence of cell type fraction estimates, one could have used either the random guess or the non-negative matrix factorization approach [65]. The difficulty in such approaches, however, is in the ambiguity of the association of the biological cell types with the estimated profiles. We point out that a subtle distinction should in principle be made between the cell type fractions and cell type contributions to overall gene expression, as different cell types could produce different amount of mRNA per cell; what we use in our approach is cell type contributions, and we assume that cell type fractions are similar.

#### Mapping of microarray probe sets to UCSC known genes

We compared our classification results to those in previously published studies. These studies utilized microarrays for gene expression measurements. Schultz et al. [8] and Shima et al. [9] used Affymetrix MGU74 A,B,C v2 microarrays while Chalmel et al. [6] used MG430 2.0 microarrays. To find a correspondence between our UCSC gene names and the MGU74 probesets we use the knownToAffyU74 table from the UCSC genome browser database. Chalmel et al. [6] provide Ensembl gene names for their probesets, so we could use knownToEnsembl in this case to determine the correspondence.

#### Analysis of RNA-Seq from cell-sorted samples

Soumillon et al. [13] performed RNA-Seq of sorted cell populations. Gene expression values for the five sorted cell types were downloaded from Gene Expression Omnibus, accession number GSE43717. Genes were clustered into five corresponding clusters using k-means with a cosangle distance measure, as described above. Lists of genes in the four clusters defined in [13] were obtained from the authors. Conversion from Ensembl gene names to gene symbols was done with the biomaRt package in R.

#### Construction of splices

Because our data consists of short (36 bp, trimmed to 32 bp – see above) single-end reads, effective *de novo* splicing discovery is unfeasible. Therefore, we looked for potential splicing events between non-neighboring exons within each gene, for all genes. In search of novel splices we adopted the following strategy. All known isoforms of a gene contribute to its set of exons, and each pair of non-overlapping non-neighboring exons produced a candidate splice. We note that for known splices, only neighboring exons of each annotated isoform are considered. 28 bp fragments from each exon were merged, in order to guarantee at least 4 bases present on either side of the splice (as we use 32 bp reads). A handful of cases of exons shorter than 28 bp resulted in a splice interval shorter than 56 bp. Replicates have been removed. In total, over 2 million splice intervals have been constructed, and all the reads have been aligned to the extended genome consisting of the chromosome sequences plus the constructed set of splice intervals. If a read did not align to the genome or a known splice, but aligned to an alternative splice (thus skipping one or more exons in known isoforms, or else forming an inter-known-isoform splice) it was considered as a candidate alternative splicing event.

#### Polyadenylation

As in the case of novel alternative splicing, we took into account the nature of our short single-end read library. To identify polyadenylation start sites, we considered reads that did not align to the genome or transcriptome as the candidate reads covering such sites. Candidate reads were expected to have either the [ACGT]_n_A_36-n_ composition, for the transcript strand, or the T_36-n_[ACGT]_n_ composition, for the opposite strand. The non-aligned reads were mapped to the genome by selecting sub-reads of varying lengths, skipping bases either at the end or at the beginning of the read, and finding the longest possible alignment (the [ACGT]_n_ part). Skipping bases at the beginning assumes that the read may be from a strand complementary to the mRNA and hence its 5′ start corresponds to the 3′ poly-A mRNA tail, while skipping bases at the 3′ end of the read assumes that it is on the same strand as the mRNA. Each candidate position had to have a polyadenylation signal sequence ANTAAA within 50 bases upstream of it (in the transcript orientation), and the non-aligning part of the read had to be enriched for A’s (at the end of the read, for the transcript strand) or T’s (at the beginning of the read, for the complementary strand), consisting of >75% of these bases. This threshold was lower than 100% to allow for sequencing errors in typically very short poly-A read stretches.

## Abbreviations

Aa: Amino acid; Dpp: Days post partum; lincRNA: Large intergenic/intervening non-coding RNA; MSCI: Meiotic sex chromosome inactivation; ORF: Open reading frame; piRNA: PIWI-interacting RNA; Pol II: RNA polymerase II; RPKM: Reads per kilobase per million of reads; TSS: Transcription start site.

## Competing interests

The authors declare that they have no competing interests.

## Authors’ contributions

GM analyzed data, developed and implemented deconvolution algorithm, and wrote the manuscript. PPK prepared mRNA-Seq libraries and analyzed data. JK prepared PolII ChIP-Seq libraries. MAB prepared RNA samples. RDCO supervised the study. All authors participated in study design. All authors read and approved the final manuscript.

## Supplementary Material

Additional file 1 **Contains Tables S1, S2, S3, S4, S5, S6, S7, S8 and S9, Figures S1, S2, S3, S4, S5, S6, S7, S8, S9, S10, S11, S12, S13, S14, S15, S16 and S17 and legends.** Click here for file

Additional file 2 Is a table listing the temporal gene expression (RPKM) calculated from the mRNA-Seq data and the temporal gene expression clustering. Click here for file

Additional file 3 Is a table listing 1048 protein-coding genes identified as meiotically expressed, which have not been classified by previous microarray studies. Click here for file

Additional file 4 Is a table listing cell type-specific gene expression (RPKM) estimates obtained from the deconvolution calculations and the cell type-specific gene expression clustering. Click here for file

Additional file 5 Is a table listing novel splice junctions. Click here for file

Additional file 6 Is a table listing predicted Ensembl gene models that have some support in our expression data. Click here for file

Additional file 7 Is a table listing predicted Genscan gene models that have some support in our expression data. Click here for file

Additional file 8 Is a table listing 59 lincRNA regions not overlapping UCSC known genes displaying variable and high expression through the time course of spermatogenesis. Click here for file

Additional file 9 Contains protein sequences of two predicted isoforms at genomic locus chr7:19684394-19699830. Click here for file

## References

[B1] Cheng C Mruk D The biology of spermatogenesis: the past, present and future Phil Trans R Soc B 2010 15 1459 1463 10.1098/rstb.2010.0024 20403863 PMC2871927

[B2] Calvel P Rolland A Jégou B Pineau C Testicular postgenomics: targeting the regulation of spermatogenesis Phil Trans R Soc B 2010 15 1481 1500 10.1098/rstb.2009.0294 20403865 PMC2871924

[B3] Geisinger A Rodríguez-Casuriaga R Flow cytometry for gene expression studies in mammalian spermatogenesis Cytogenet Genome Res 2010 15 46 56 10.1159/000291489 20389037

[B4] Dym M Kakkinaki M He Z Spermatogonial stem cells: mouse and human comparisons Birth Defects Research (Part C) 2009 15 27 34 10.1002/bdrc.20141 19306345

[B5] Phillips BT Cassei K Orwig KE Spermatogonial stem cell regulation and spermatogenesis Phil Trans R Soc B 2010 15 1663 1678 10.1098/rstb.2010.0026 20403877 PMC2871929

[B6] Chalmel F Rolland AD Niederhauser-Wiederkehr C Chung SSW Demougin P Gattiker A Moore J Patard J-J Wolgemuth DJ Jegou B Primig M The conserved transcriptome in human and rodent male gametogenesis Proc Natl Acad Sci USA 2007 15 20 8346 8351 10.1073/pnas.0701883104 17483452 PMC1864911

[B7] Fallahi M Getun IV Wu ZK Bois PRJ A global expression switch marks pachytene initiation during mouse male meiosis Genes 2010 15 469 483 10.3390/genes1030469 24710097 PMC3966219

[B8] Schultz N Hamra FK Garbers DL A multitude of genes expressed solely in meiotic or postmeiotic spermatogenic cells offers a myriad of contraceptive targets Proc Natl Acad Sci USA 2003 15 21 12201 12206 10.1073/pnas.1635054100 14526100 PMC218736

[B9] Shima JE McLean DJ McCarrey JR Griswold MD The murine testicular transcriptome- characterizing gene expression in the testis during the progression of spermatogenesis Biol Reprod 2004 15 319 330 10.1095/biolreprod.103.026880 15028632

[B10] Harr B Turner L Genome-wide analysis of alternative splicing evolution among Mus subspecies Mol Ecol 2010 15 1 228 239 20331782 10.1111/j.1365-294X.2009.04490.x

[B11] Mouse ENCODE transcriptome data http://genome.ucsc.edu/cgi-bin/hgTrackUi?db=mm9&g=wgEncodeCshlLongRnaSeq (NCBI’s Gene Expression Omnibus accessions GSM900193 and GSM929715)

[B12] Laiho A Kotaja N Gyenesei A Sironen A Transcriptome profiling of the murine testis during the first wave of spermatogenesis PLoS One 2013 15 14 e61558 23613874 10.1371/journal.pone.0061558 PMC3629203

[B13] Soumillon M Necsulea A Weier M Brawand D Zhang X Gu H Pauline B Kokkinaki M Nef S Gnirke A Dym M de Massy B Mikkelsen TS Kaessmann H Cellular source and mechanisms of high transcriptome complexity in the mammalian testis Cell Reports 2013 15 2179 2190 10.1016/j.celrep.2013.05.031 23791531

[B14] Wang Z Gerstein M Snyder M RNA-Seq: a revolutionary tool for transcriptomics Nat Rev Genet 2009 15 1 57 63 10.1038/nrg2484 19015660 PMC2949280

[B15] Matlin A Clark F Smith C Understanding alternative splicing: towards a cellular code Nat Rev Mol Cell Biol 2005 15 5 386 398 10.1038/nrm1645 15956978

[B16] Barash Y Calarco J Gao W Pan Q Wang X Shai O Blencowe B Frey B Deciphering the splicing code Nature 2010 15 7294 53 59 10.1038/nature09000 20445623

[B17] Werner T Next generation sequencing allows deeper analysis and understanding of genomes and transcriptomes including aspects to fertility Reprod Fertil Dev 2011 15 1 75 80 10.1071/RD10247 21366983

[B18] Fleischer J Breer H Strotmann J Mammalian olfactory receptors Front Cell Neurosci 2009 15 9 19753143 10.3389/neuro.03.009.2009 PMC2742912

[B19] Kratz E Dugas JC Ngai J Odorant receptor gene regulation: implications from genomic organization Trends Genet 2002 15 1 29 34 10.1016/S0168-9525(01)02579-3 11750698

[B20] Yoshida K Kondoh G Matsuda Y Habu T Nishimune Y Morita T The mouse *RecA*-like gene *Dmc1* is required for homologous chromosome synapsis during meiosis Mol Cell 1998 15 707 718 10.1016/S1097-2765(00)80070-2 9660954

[B21] Hayashi K Yoshida K Matsui Y A histone H3 methyltransferase controls epigenetic events required for meiotic prophase Nature 2005 15 7066 374 378 10.1038/nature04112 16292313

[B22] Baudat F Buard J Grey C Fledel-Alon A Ober C Przeworski M Coop G de Massy B PRDM9 is a major determinant of meiotic recombination hotspots in humans and mice Science 2010 15 5967 836 40 10.1126/science.1183439 20044539 PMC4295902

[B23] Smagulova F Gregoretti I Brick K Khil P Camerini-Otero R Petukhova G Genome-wide analysis reveals novel molecular features of mouse recombination hotspots Nature 2011 15 7343 375 378 10.1038/nature09869 21460839 PMC3117304

[B24] La Salle S Palmer K O’Brien M Schimenti JC Eppig J Handel MA Spata22, a novel vertebrate-specific gene, is required for meiotic progress in mouse germ cells Biol Reprod 2012 15 2 45 10.1095/biolreprod.111.095752 22011390 PMC3290669

[B25] Bellvé A Cavicchia J Millette C O’Brien D Bhatnagar Y Dym M Spermatogenic cells of the prepuberal mouse: isolation and morphological characterization J Cell Biol 1977 15 1 68 85 10.1083/jcb.74.1.68 874003 PMC2109873

[B26] Lu P Nakorchevskiy A Marcotte E Expression deconvolution: a reinterpretation of DNA microarray data reveals dynamic changes in cell populations Proc Natl Acad Sci USA 2003 15 18 10370 10375 10.1073/pnas.1832361100 12934019 PMC193568

[B27] Stuart R Wachsman W Berry C Wang-Rodriguez J Wasserman L Klacansky I Masys D Arden K Goodison S McClelland M Wang Y Sawyers A Kalcheva I Tarin D Mercola D In silico dissection of cell-type-associated patterns of gene expression in prostate cancer Proc Natl Acad Sci USA 2004 15 2 615 620 10.1073/pnas.2536479100 14722351 PMC327196

[B28] Lähdesmäki H Shmulevich L Dunmire V Yli-Harja O Zhang W In silico microdissection of microarray data from heterogeneous cell populations BMC Bioinforma 2005 15 54 10.1186/1471-2105-6-54 PMC1274251 15766384

[B29] Wang M Master S Chodosh L Computational expression deconvolution in a complex mammalian organ BMC Bioinforma 2006 15 328 10.1186/1471-2105-7-328 PMC1559723 16817968

[B30] Abbas A Wolslegel K Seshasayee D Modrusan Z Clark H Deconvolution of blood microarray data identifies cellular activation patterns in systemic lupus erythematosus PLoS One 2009 15 7 e6098 10.1371/journal.pone.0006098 19568420 PMC2699551

[B31] Shen-Orr S Tibshirani R Khatri P Bodian D Staedtler F Perry N Hastie T Sarwal M Davis M Butte A Cell type-specific gene expression differences in complex tissues Nat Methods 2010 15 4 287 289 10.1038/nmeth.1439 20208531 PMC3699332

[B32] Bellvé A Millette C Bhatnagar Y O’Brien D Dissociation of the mouse testis and characterization of isolated spermatogenic cells J Histochem Cytochem 1977 15 7 480 494 10.1177/25.7.893996 893996

[B33] Goetz P Chandley A Speed R Morphological and temporal sequence of meiotic prophase development at puberty in the male mouse J Cell Sci 1984 15 249 263 6538881 10.1242/jcs.65.1.249

[B34] Namekawa SH Park PJ Zhang L-F Shima JE McCarrey JR Griswold MD Lee JT Postmeiotic sex chromatin in the male germline of mice Curr Biol 2006 15 660 667 10.1016/j.cub.2006.01.066 16581510

[B35] Waldman Ben-Asher H Shahar I Yitzchak A Mehr R Don J Expression and chromosomal organization of mouse meiotic genes Mol Reprod Dev 2010 15 241 248 19953644 10.1002/mrd.21139

[B36] Hermo L Pelletier RM Cyr DG Smith CE Surfing the wave, cycle, life history, and genes/proteins expressed by testicular germ cells. Part 1: background to spermatogenesis, spermatogonia, and spermatocytes Microsc Res Tech 2010 15 4 241 278 10.1002/jemt.20783 19941293

[B37] Guttman M Amit I Garber M French C Lin M Feldser D Huarte M Zuk O Carey B Cassady J Cabili M Jaenish R Mikkelsen T Jacks T Hacohen N Bernstein B Kellis M Regev A Rinn J Lander E Chromatin signature reveals over a thousand highly conserved large non-coding RNAs in mammals Nature 2009 15 223 227 10.1038/nature07672 19182780 PMC2754849

[B38] Guttman M Garber M Levin J Donaghey J Robinson J Adiconis X Fan L Koziol M Gnirke A Nusbaum C Rinn J Lander E Regev A Ab initio reconstruction of transcriptomes of pluripotent and lineage committed cells reveals gene structures of thousands of lincRNAs Nat Biotechnol 2010 15 503 510 10.1038/nbt.1633 20436462 PMC2868100

[B39] Guttman M Donaghey J Carey B Garber M Grenier J Munson G Young G Bergstrom Lucas A Ach R Bruhn L Yang X Amit I Meissner A Regev A Rinn J Root D Lander E lincRNAs act in the circuitry controlling pluripotency and differentiation Nature 2011 15 7364 295 300 10.1038/nature10398 21874018 PMC3175327

[B40] Shi Y Alternative polyadenylation: new insights from global analyses RNA 2012 15 2105 2117 10.1261/rna.035899.112 23097429 PMC3504663

[B41] Shepard PJ Choi E-A Lu J Flanagan LA Hertel KJ Shi Y Complex and dynamic landscape of RNA polyadenylation revealed by PAS-Seq RNA 2011 15 761 772 10.1261/rna.2581711 21343387 PMC3062186

[B42] Derti A Garrett-Engele P MacIsaac KD Stevens RC Sriram S Chen R Rohl CA Johnson JM Babak T A quantitative atlas of polyadenylation in five mammals Genome Res 2012 15 1173 1183 10.1101/gr.132563.111 22454233 PMC3371698

[B43] Turner J Meiotic sex chromosome inactivation Development 2007 15 10 1823 1831 10.1242/dev.000018 17329371

[B44] Vaskova E Pavlova S Shevchenko A Zakinian S Meiotic inactivation of sex chromosomes in mammals Russ J Genet 2010 15 4 385 393 10.1134/S1022795410040010 20536013

[B45] Khil PP Smirnova NA Romanienko PJ Camerini-Otero RD The mouse X chromosome is enriched for sex-biased genes not subject to selection by meiotic sex chromosome inactivation Nat Genet 2004 15 642 646 10.1038/ng1368 15156144

[B46] Shen Y Yue F McCleary DF Ye Z Edsall L Kuan S Wagner U Dixon J Lee L Lobanenkov VV Ren B A map of cis-regulatory sequences in the mouse genome Nature 2012 15 116 120 10.1038/nature11243 22763441 PMC4041622

[B47] Sun H Wu J Wickramasinghe P Pal S Gupta R Bhattacharyya A Agosto-Perez FJ Showe LC Huang TH-M Davulugri RV Genome-wide mapping of RNA Pol-II promoter usage in mouse tissues by ChIP-seq Nucleic Acids Res 2011 15 1 190 201 10.1093/nar/gkq775 20843783 PMC3017616

[B48] Kawaji H Severin J Lizio M Waterhouse A Katayama S Irvine KM Hume DA Forrest AR Suzuki H Carninci P Hayashizaki Y Daub CO The FANTOM web resource: from mammalian transcriptional landscape to its dynamic regulation Genome Biol 2009 15 4 R40 10.1186/gb-2009-10-4-r40 19374775 PMC2688931

[B49] Brick K Smagulova F Khil P Camerini-Otero R Petukhova G Genetic recombination is directed away from functional genomic elements in mice Nature 2012 15 642 645 10.1038/nature11089 22660327 PMC3367396

[B50] Huang D Sherman B Lempicki R Systematic and integrative analysis of large gene lists using DAVID bioinformatics resources Nature Protoc 2009 15 1 44 57 10.1038/nprot.2008.211 19131956

[B51] Hosack D Glynn D Sherman B Lane H Lempicki R Identifying biological themes within lists of genes with EASE Genome Biol 2003 15 10 R70 10.1186/gb-2003-4-10-r70 14519205 PMC328459

[B52] Muse G Gilchrist D Nechaev S Shah R Parker J Grissom S Zeitlinger J Adelman K RNA polymerase is poised for activation across the genome Nat Genet 2007 15 12 1507 1511 10.1038/ng.2007.21 17994021 PMC2365887

[B53] Nechaev S Adelman K Pol II waiting in the starting gates: regulating the transition from transcription initiation into productive elongation Biochim Biophys Acta 1809 15 34 45 10.1016/j.bbagrm.2010.11.001 PMC3021596 21081187

[B54] Mueller J Mahadevaiah S Park P Warburton P Page D Turner J The mouse X chromosome is enriched for multicopy testis genes showing postmeiotic expression Nat Genet 2008 15 6 794 799 10.1038/ng.126 18454149 PMC2740655

[B55] Lau N Seto A Kim J Kuramochi-Miyagawa S Nakano T Bartel D Kingston R Characterization of the piRNA complex from rat testes Science 2006 15 363 367 10.1126/science.1130164 16778019

[B56] Siomi M Sato K Pezic D Aravin A PIWI-interacting small RNAs: the vanguard of genome defence Nature 2011 15 246 258 10.1038/nrm3089 21427766

[B57] Gan H Lin X Zhang Z Zhang W Liao S Wang L Han C piRNA profiling during specific stages of mouse spermatogenesis RNA 2011 15 1191 1203 10.1261/rna.2648411 21602304 PMC3138557

[B58] Bellani MA Boateng KA McLeod D Camerini-Otero RD The expression profile of the major mouse SPO11 isoforms indicates that SPO11beta introduces double strand breaks and suggests that SPO11alpha has an additional role in prophase in both spermatocytes and oocytes Mol Cell Biol 2010 15 18 4391 4403 10.1128/MCB.00002-10 20647542 PMC2937527

[B59] Mortazavi A Williams BA McCue K Schaeffer L Wold B Mapping and quantifying mammalian transcriptomes by RNA-Seq Nat Methods 2008 15 7 621 628 10.1038/nmeth.1226 18516045 PMC13303166

[B60] Zhang Y Liu T Meyer C Eeckhoute J Johnson D Bernstein B Nusbaum C Myers R Brown M Li W Liu X Model-based analysis of ChIP-Seq (MACS) Genome Biol 2008 15 R137 10.1186/gb-2008-9-9-r137 18798982 PMC2592715

[B61] Quinlan AR Hall IM BEDTools: a flexible suite of utilities for comparing genomic features Bioinformatics 2010 15 6 841 842 10.1093/bioinformatics/btq033 20110278 PMC2832824

[B62] Xu H Handoko L Wei X Ye C Sheng J Wei C Lin F Sung W A signal-noise model for significance analysis of ChIP-seq with negative control Bioinformatics 2010 15 9 1199 1204 10.1093/bioinformatics/btq128 20371496

[B63] van der Laan MJ Pollard KS A new algorithm for hybrid hierarchical clustering with visualization and the bootstrap Journal of Statistical Planning and Inference 2003 15 275 303 10.1016/S0378-3758(02)00388-9

[B64] Eisen MB Spellman PT Brown PO Botstein D Cluster analysis and display of genome-wide expression patterns Proc Natl Acad Sci USA 1998 15 14863 14868 10.1073/pnas.95.25.14863 9843981 PMC24541

[B65] Gaujoux R Seoighe C A flexible R package for nonnegative matrix factorization BMC Bioinforma 2010 15 367 10.1186/1471-2105-11-367 PMC2912887 20598126

