# Supplementary material for: Integrated transcriptome analysis of mouse spermatogenesis
Source: BMC Genomics. 2014 Jan 18;15:39. doi: 10.1186/1471-2164-15-39 (PMC3906902; doi:10.1186/1471-2164-15-39)
Supplement: Additional file 1 — Contains Tables S1, S2, S3, S4, S5, S6, S7, S8 and S9, Figures S1, S2, S3, S4, S5, S6, S7, S8, S9, S10, S11, S12, S13, S14, S15, S16 and S17 and legends. [file 1471-2164-15-39-S1.pdf]

List of Supplementary Tables

Table S1. Sequencing yield of individual samples. Read counts are given in millions of reads. Total - total number of reads past Quality Control (QC); Aligned - number of reads past quality filtering (PF) aligned to genome; Unique - number of reads PF, aligned to a single location in the genome; Distinct - number of reads after removal of potential PCR duplicates in the Unique column; Splices - number of distinct reads past quality filtering, uniquely aligned to known splices.

Table S2. Detection of known coding genes, and of known isoforms of multi-isoform coding genes. We see about 85% of coding genes in each sample, and about 70% of detectable isoforms. Since not all isoforms possess an exon or splice junction unique to a given isoform (each feature can be shared by two or more different isoforms), not all isoforms are detectable. By "hits" we mean distinct reads falling within the gene transcript, and "feature" can be either an exon or splice junction unique to a given gene isoform. The "splicing" row provides a count of genes in which splicing is observed.

Table S3. Expression clusters of selected genes associated with meiosis and/or spermatogenesis (cf. Table 1). This list of genes was constructed from genes associated with either the Gene Ontology (GO) term "meiosis (GO:0007126)" or "spermatogenesis (GO:0007283)" and their children GO categories. For each gene in the list, the rightmost column indicates the term(s) associated with that gene. GO was accessed through the http://www.geneontology.org/ website.

Table S4. Deconvolution classification of gene expression by cell type. For a selection of genes, we show the cell types with estimated non-zero expression, for different iterations and significance cutoffs.

Table S5. Detection of known exon-exon splices. There are 205151 known splices, based on known gene isoforms, and about 75% of them are detected in our data. When demanding ≥2 hits, only distinct hits qualify.

Table S6. Detection of novel splices based on known isoforms. There are 2065232 (above $2\cdot10^6$) possible splices – see text for details. We see about 13000 novel exon-exon splices in total. When demanding ≥2 hits, only distinct hits qualify.

Table S7. Overlaps of Pol II peaks at three time points with various marks and genomic features.

Table S8. Consistency of our deconvolution cell type assignments: comparison of the 5, 6 and 7 cell-type calculations. In total, 14259 genes were included in deconvolution. In the first six rows, "within each of (5, 6, 7)" indicates comparisons made within calculations with identical cell types at different iterations, but for all three cell type numbers. For example, in the first row, a gene having identical expression presence in iterations 1 and 3 in 5 cell-type calculation with p-value cutoff of 0.05, and having identical (but possibly different from a 5 cell-type) expression presence in iterations 1 and 3 in 6 cell-type calculation with the same p-value cutoff, and similarly for 7 cell-type calculation, will contribute one count. The remaining rows compare all of the indicated cell type-number calculations, at one or two iterations and indicated p-value cutoffs.

Table S9. Tables of Pearson and Spearman correlation coefficients for each considered cell type, between different iterations (1, 3 and 10), as indicated in the left columns. Here we consider 5- and 6-cell type results. In the 6-cell type analysis, type B is split into Bs (types A and B spermatogonia) and Bl (pre-leptotene and leptotene spermatocytes). For 5 vs. 6 cell types tables, the first (left) iteration is for 5-cell type, and the second (right) is for 6-cell type calculations. We find a good persistence of results from iteration to iteration. Given the similar cell type fraction values for types Bs and Bl these two types are by far the least stable in the 6-cell type analysis.

List of Supplementary Figures

Figure S1. Read coverage along the length of expressed transcripts. Coverage is largely uniform with a slight decrease towards the 5’ end.

Figure S2. Comparison of our temporal expression clusters (see Figure 2) with three previously published gene expression studies [6], [8] and [9]. Chalmel et al. report 4 clusters – somatic, mitotic, meiotic and post-meiotic, while Shima et al. have 5 clusters A-E. In case of Schultz et al. we present our clustering of their microarray data. Each cell gives the number of genes common to one of our clusters and one of the clusters from the three papers. There is a reasonable agreement between various clustering results, and it is seen that our clusters 4, 6 and 7 mostly correspond to the meiotic cluster defined in Chalmel et al., to cluster D of Shima et al. and to clusters 5 and 7 derived from Schultz et al. data.

Figure S3. Comparisons of our deconvolution estimates and temporal clusters with the cell-sorted RNA-Seq results of Soumillon et al. [13]. (A) Scatter plots of log-transformed gene expression + 0.1 RPKM (a small value 0.1 is added to visualize zero expression on a log-log plot). A-E are values estimated in our respective deconvolution cell types; sl, sg, sc, sd and sz are Sertoli, spermatogonia, spermatocytes, spermatids and spermatozoa measurements from [13]. Numbers are Spearman correlation values. (B) Normalized gene expression heatmap for [13], ordered by 5 k-means clusters (our clustering of 18,411 genes from [13]). (C) Comparison of our deconvolution clusters with our clustering of data from [13] (cf. B). Each cell has the number of genes belonging to the respective deconvolution and experimental clusters. (D) Comparison of our deconvolution clusters with the four clusters defined in [13]. (E) and (F) Same as (C) and (D), but for our temporal clusters.

Figure S4. Histograms of predicted gene expression by cell type. n – number of genes found to be expressed in a given cell type, m – missing genes (having zero expression). Out of 14259 genes selected for deconvolution analysis, 12300 genes are predicted expressed in somatic cells (A) and 7609 are predicted to be expressed in secondary spermatocytes and/or spermatids (E). The expression signatures of other cell types are more specific and the majority of genes are predicted to be silent (or having insignificant expression) in them.

Figure S5. Comparison of our temporal clustering of gene expression with cell type-specific gene expression calculations after 10 iterations. In the deconvolution results, gene expression fraction in each cell type, A through E, is defined as the fraction of total gene expression (the sum over all cell types). Each number above is the sum of gene expression fractions for a given cell type, for genes that belong to the indicated temporal expression cluster on the abscissa. There is an overall agreement between the two analyses. In addition, a noticeable contribution to expression in post-meiotic cell type E comes from all eight temporal clusters. This indicates that there is a number of genes expressed mostly post-meiotically, but in other cell types as well, at lower levels. Given that post-meiotic cell types are practically absent until after 20dpp, such genes can fall in different temporal clusters.

Figure S6. Distribution of the number of skipped exons in novel splices. In the case of multiple choices, we pick the smaller number. The majority (between 85 to 90%, except at 16dpp with about 77%) of novel splices have one exon skipped.

Figure S7. Chromosomes X, 3, 7 and 17 have statistically significant deviations in proportions of genes split by temporal expression cluster (black bars), when compared with all autosomes (light grey bars). Chromosome X is by far the most obvious outlier - due to MSCI, there are no X-linked genes in clusters 6 and 7, and they are strongly underrepresented in clusters 4 and 5. On the other hand, temporal clusters 2 and 3, which contain pre-meiotic and early meiotic genes (cf. Figure S4), are enriched on X.

Figure S8. Spearman correlation of TSS-proximal 500bp (red) and TSS-distal/gene body (blue) Pol II read coverage with gene expression at different time points (abscissa). Pol II at 10dpp (top), Pol II at 16dpp (middle), Pol II in adult sample (bottom) are shown (See Materials and Methods for details). Gene body correlation for Pol II at 16dpp is higher than TSS-proximal correlation because in this Pol II sample we used phosphorylated Serine-5 specific antibody, which yielded more signal along transcribed genes.

Figure S9. Venn diagram of the proportion of TSS-proximal Pol II peaks at 10dpp (red), 16dpp (green) and adult (blue) among all genes. 51% of 27,775 TSS intervals have peak at one or more time points. Note that 37% of the intervals belong to genes with low expression throughout the measured course of spermatogenesis; these genes mostly don't have Pol II peaks at their TSS's – see Figure S8.

Figure S10. Venn diagrams of the proportions of TSS-proximal Pol II peaks at 10dpp (red), 16dpp (green) and adult (blue), split by gene expression cluster (EC). EC 1 through 8 are the expression clusters discussed in the paper (early to late), ‘const’ are genes with maximal RPKM above 2 but the fold change less than 2 over the whole time course, and ‘low’ are the genes with maximal RPKM below 2 at all time points. The percentage for each cluster is the percent of genes considered in that cluster that have at least one peak at any of their TSS’s at any time point. For each cluster separately, circle sizes and overlaps are drawn to scale. Cluster sizes are 1215, 2875, 2437, 1031, 881, 733, 711, 1179, 1444, and 7352 for ECs 1, 2, 3, 4, 5, 6, 7, 8, ‘const’ and ‘low’, respectively.

Figure S11. Annotated model of Tex16 gene possibly misses long, ~200kb 5’ part. We can detect both the presence of potential exons and Pol II signal upstream of the available annotation.

Figure S12. Enriched Gene Ontology (GO) and INTERPRO categories in novel meiotic genes in various functional annotation databases. The data is obtained from the DAVID Functional Annotation Tool [50].

Figure S13. Example of piRNA cluster on chromosome 7. The cluster interval is based on [55] (red bar), and its coordinates are chr7:77,023,159-77,099,158. There is no RefSeq annotation in this region, however there are multiple short gene transcripts annotated in UCSC knownGene. Short gene transcripts on the forward and reverse strand are shown in red and blue colors, respectively. mRNA signal starts to be noticeable at around 14dpp, and there is a corresponding Pol II signal at 16dpp. There is no Pol II signal at 10dpp, and there is a weaker signal in the adult sample. Note that in this and some other examples there is a distinguishable Pol II peak marking the divergent transcription exactly in agreement with the UCSC annotation.

Figure S14. Initial and corrected significance values of F-statistics (testing for non-randomness of deconvolution algorithm predictions). BH – Benjamini-Hochberg, BY – Benjamini-Yekutieli procedures. We note that these estimates do not take the stochastic nature of our algorithm into account. Nevertheless, they should provide certain confidence in the statistical significance of our results.

Figure S15. Comparison of genes predicted by our deconvolution analysis to lie in clusters corresponding to cell types A-E (after 10 iterations; cf. Figure 4) with experimental cell type sorted results of Chalmel et al. [6]. SO, MI, ME, PM stand for somatic, mitotic, meiotic and post-meiotic clusters.

Figure S16. Comparison of observed and reconstructed gene expression datasets after 1 iteration (i.e., with the initial estimate of cell type fractions). Pearson and Spearman correlation coefficients are given. Numbers of genes with zero expression for observed, reconstructed and both datasets are provided in square brackets as well. Reconstructed temporal gene expression is based on cell type contributions at different dpp, and on the estimated cell type-specific gene expressions.

Figure S17. Comparison of observed and reconstructed gene expression datasets after 10 iterations. Pearson and Spearman correlation coefficients are given. Numbers of genes with zero expression for observed, reconstructed and both datasets are provided in square brackets as well. In comparison to Figure S15 for iteration 1, a better agreement is found here, especially at 38dpp.

Sample	Total	Aligned	Unique	Distinct	Splices
6dpp	47	27	17	6.2	0.42
10dpp	64	27	12	6.7	0.39
12dpp	42	22	13	6.1	0.39
14dpp	44	22	13	6.6	0.44
16dpp	96	54	31	6.4	0.42
18dpp	82	41	23	9.8	0.61
20dpp	46	25	15	6.2	0.41
38dpp	65	34	21	5.5	0.43

Table S1. Sequencing yield of individual samples. Read counts are given in millions of reads. Total - total number of reads past Quality Control (QC); Aligned - number of reads past quality filtering (PF) aligned to genome; Unique - number of reads PF, aligned to a single location in the genome; Distinct - number of reads after removal of potential PCR duplicates in the Unique column; Splices - number of distinct reads past quality filtering, uniquely aligned to known splices.

Total known genes: 27389

Total known isoforms: 49409

Total known coding genes: 20567

Total known coding isoforms: 39481

1-exon coding isoforms: 2058

multi-exon coding isoforms: 37423

Coding genes having multiple isoforms: 10205

Isoforms in multi-isoform coding genes: 31631 (29037 of them are multi-exon)

Sample	*6dpp*	*10dpp*	*12dpp*	*14dpp*	*16dpp*	*18dpp*	*20dpp*	*38dpp*
**Detection of known coding genes (out of total 20567)**								
hits ≥1	17287	17554	17352	17535	17511	17771	17471	17578
hits ≥2	16691	16964	16729	16893	16916	17230	16881	16993
splicing	11758	10801	10583	10929	10743	11289	10520	10808
**Detection of all known isoforms for multi-isoform coding genes (total 31631; detectable 18345)†**								
uniq feature hits ≥1	9337	8860	8943	9230	9061	9681	8578	8742
uniq feature hits ≥2	7159	6561	6834	7005	7081	7604	6525	6902

†we look at the number of distinct hits to a feature (exon or splice) unique to a given isoform.

Table S2. Detection of known coding genes, and of known isoforms of multi-isoform coding genes. We see about 85% of coding genes in each sample, and about 70% of detectable isoforms. Since not all isoforms possess an exon or splice junction unique to a given isoform (each feature can be shared by two or more different isoforms), not all isoforms are detectable. By "hits" we mean distinct reads falling within the gene transcript, and "feature" can be either an exon or splice junction unique to a given gene isoform. The "splicing" row provides a count of genes in which splicing is observed.

Gene	Temporal 1-8	Schultz'03 1-8	Shima'04 A-E	Chalmel'07 SO,MI,ME,PM	Deconvolution A-E	Biological Process (GO)
1110017D15Rik	8	8	D	PM	E	spermatogenesis
2410076I21Rik	2	-	-	MI	A	meiosis
4632434I11Rik	6	6	D	ME	D	spermatogenesis
4932438A13Rik	4	-	-	-	C	spermatogenesis
Ace	8	6	-	PM	E	spermatogenesis
Acox1	3	3	-	SO	B	spermatogenesis
Acrbp	7	7	D	ME	E	spermatogenesis
Acsbg2	8	8	E	PM	E	spermatogenesis
Acvr2a	3	6	-	-	B	spermatogenesis
Adad1	6	8	D	ME	C	spermatogenesis
Adam18	7	7	D	ME	D	spermatogenesis
Adam1b	7	7	-	ME	D	spermatogenesis
Adam24	8	-	-	PM	E	spermatogenesis
Adam25	8	-	-	PM	E	spermatogenesis
Adam26a	8	-	-	PM	E	spermatogenesis
Adamts2	2	-	A	SO	A	spermatogenesis
Adcyap1r1	1	-	-	SO	A	spermatogenesis
Adrm1	2	5	-	-	C	spermatogenesis
Aff4	3	4	-	-	C	spermatogenesis
Alkbh5	3	3	-	-	C	spermatogenesis
Alms1	4	-	-	ME	B	spermatogenesis
Apitd1	-	5	-	-	B	meiosis
Apob	-	-	-	-	-	spermatogenesis
Ar	2	3	-	-	B	spermatogenesis
Aspm	3	2	-	-	B	spermatogenesis
Asz1	4	-	-	ME	C	meiosis, spermatogenesis
Aurka	7	7	D	-	D	meiosis
Aurkc	-	-	-	ME	-	meiosis
Axl	2	-	-	MI	A	spermatogenesis
Azi1	5	7	D	ME	D	spermatogenesis
B4galnt1	3	5	-	ME	B	spermatogenesis
Bax	2	3	B	SO	A	spermatogenesis
Bbs2	4	6	D	ME	C	spermatogenesis
Bbs4	-	-	D	-	D	spermatogenesis
Bcl2l1	4	5	D	-	C	spermatogenesis
Bcl2l11	3	3	C	MI	B	spermatogenesis
Bcl6	2	3	-	-	B	spermatogenesis
Bik	3	-	-	-	C	spermatogenesis
Bmp8a	-	-	-	-	-	spermatogenesis
Bmp8b	-	-	-	-	-	spermatogenesis
Boll	6	-	-	ME	C	meiosis, spermatogenesis
Brca2	3	6	-	-	B	meiosis, spermatogenesis
Brdt	6	6	D	ME	C	meiosis, spermatogenesis
Bub3	-	-	-	-	C	meiosis

Gene	Temporal 1-8	Schultz'03 1-8	Shima'04 A-E	Chalmel'07 SO,MI,ME,PM	Deconvolution A-E	Biological Process (GO)
Cabyr	8	-	-	PM	E	spermatogenesis
Cadm1	3	4	-	MI	B	spermatogenesis
Calca	-	-	-	-	-	spermatogenesis
Calr	-	-	-	-	C	meiosis
Calr3	8	8	E	PM	E	spermatogenesis
Camk2b	2	-	-	-	A	meiosis
Capza3	8	8	-	PM	E	spermatogenesis
Catsper1	8	8	E	PM	E	spermatogenesis
Catsper2	7	7	D	ME	D	spermatogenesis
Catsper3	8	-	-	PM	E	spermatogenesis
Catsper4	8	-	-	-	E	spermatogenesis
Ccdc135	7	-	-	-	D	spermatogenesis
Ccdc33	8	-	-	PM	E	spermatogenesis
Ccin	8	-	-	PM	E	spermatogenesis
Ccna1	7	7	D	ME	E	meiosis, spermatogenesis
Ccnb3	3	-	-	-	B	meiosis
Cdc20	4	5	D	-	D	meiosis
Cdc25b	1	2	A	MI	A	meiosis
Cdk2	-	3	C	MI	B	meiosis
Cftr	6	6	-	ME	C	spermatogenesis
Cit	4	-	-	ME	C	spermatogenesis
Cks2	4	5	D	MI	C	meiosis
Clasp2	3	-	-	MI	B	meiosis
Cldn11	3	3	C	SO	B	spermatogenesis
Clgn	6	7	D	ME	D	meiosis, spermatogenesis
Cpeb1	3	-	-	-	B	meiosis
Creb3l4	8	8	E	PM	E	spermatogenesis
Crem	7	6	-	ME	E	spermatogenesis
Cyp26b1	2	-	-	MI	A	meiosis, spermatogenesis
D1Pas1	6	8	-	ME	C	spermatogenesis
Dazap1	4	2	-	-	C	spermatogenesis
Dazl	3	4	-	ME	B	meiosis, spermatogenesis
Ddx25	7	7	-	ME	D	spermatogenesis
Ddx4	6	6	D	ME	C	meiosis, spermatogenesis
Dhh	1	-	A	SO, MI	A	spermatogenesis
Dld	4	3	-	-	B	spermatogenesis
Dmc1	3	4	-	PM	B	meiosis, spermatogenesis
Dmrt1	1	3	C	MI	A	meiosis, spermatogenesis
Dmrtc2	6	-	-	ME	C	meiosis, spermatogenesis
Dnaja1	3	2	-	MI	C	spermatogenesis
Dnmt3a	2	2	A	MI	A	spermatogenesis
Dpep3	6	7	D	ME	C	meiosis
Dzip1	7	-	-	ME	E	spermatogenesis
Ehmt2	5	5	E	-	E	meiosis, spermatogenesis

Gene	Temporal 1-8	Schultz'03 1-8	Shima'04 A-E	Chalmel'07 SO,MI,ME,PM	Deconvolution A-E	Biological Process (GO)
Eif4g3	6	3	D	-	C	meiosis, spermatogenesis
Ercc1	5	-	-	-	C	spermatogenesis
Ercc4	7	7	D	ME	D	meiosis
Ereg	-	-	-	SO	-	meiosis
Espl1	4	5	D	ME	C	meiosis
Etv5	2	3	-	MI	A	spermatogenesis
Exo1	3	3	-	-	B	meiosis
Fanca	3	-	-	-	A	meiosis
Fancd2	-	5	D	ME	B	meiosis
Fancg	6	5	D	ME	C	spermatogenesis
Fancm	3	-	-	-	B	meiosis
Fbxo43	6	5	D	-	D	meiosis
Fbxo5	2	2	A	MI	A	meiosis
Fkbp6	3	3	C	ME	C	spermatogenesis
Fmn2	-	-	-	-	-	meiosis
Fndc3a	4	6	D	-	D	spermatogenesis
Foxa3	-	7	-	-	-	spermatogenesis
Fscn3	8	8	E	PM	E	spermatogenesis
Fshr	2	3	E	-	B	spermatogenesis
Fzr1	5	5	D	ME	D	meiosis
Gal3st1	3	-	-	-	C	spermatogenesis
Gamt	2	3	B	MI	C	spermatogenesis
Gata4	1	1	B	SO, MI	A	spermatogenesis
Ggn	8	7	-	-	E	spermatogenesis
Ggnbp1	7	7	-	ME	E	spermatogenesis
Ggnbp2	6	6	D	-	D	spermatogenesis
Ggt1	-	-	-	-	-	spermatogenesis
Gli1	1	1	-	-	A	spermatogenesis
Gmcl1	6	5	D	ME	C	spermatogenesis
Golga3	6	7	D	-	C	spermatogenesis
Gopc	4	6	-	-	D	spermatogenesis
Gpr3	-	-	-	-	-	meiosis
Gpx4	7	7	D	-	E	spermatogenesis
Gsr	2	3	B	MI	A	spermatogenesis
H1fnt	8	8	E	-	E	spermatogenesis
H1foo	-	-	-	-	-	meiosis
H2afx	4	5	D	-	C	meiosis, spermatogenesis
Herc2	3	3	-	-	B	spermatogenesis
Herc4	-	3	-	-	B	spermatogenesis
Hfm1	4	-	-	-	C	meiosis
Hils1	8	8	E	PM	E	spermatogenesis
Hist1h1a	3	-	-	-	C	spermatogenesis
Hist1h1t	6	6	-	-	D	spermatogenesis
Hist1h2ba	4	7	E	-	C	spermatogenesis

Gene	Temporal 1-8	Schultz'03 1-8	Shima'04 A-E	Chalmel'07 SO,MI,ME,PM	Deconvolution A-E	Biological Process (GO)
Hmga1	5	-	-	-	E	spermatogenesis
Hmga2	6	-	-	SO	D	meiosis, spermatogenesis
Hmgb2	3	3	-	MI	C	spermatogenesis
Hook1	5	6	-	-	E	spermatogenesis
Hormad1	4	-	-	ME	C	meiosis, spermatogenesis
Hormad2	4	-	E	-	C	meiosis
Hsf1	-	-	-	-	C	meiosis, spermatogenesis
Hsf2	3	8	E	-	B	spermatogenesis
Hspa1l	8	8	E	PM	E	spermatogenesis
Hspa2	6	7	D	ME	C	meiosis, spermatogenesis
Ift81	4	6	D	-	C	spermatogenesis
Immp2l	7	7	D	ME	D	spermatogenesis
Ing2	3	3	-	-	B	meiosis, spermatogenesis
Inpp5b	3	3	-	MI	B	spermatogenesis
Insl6	7	7	D	ME	D	spermatogenesis
Jag2	2	3	-	-	C	spermatogenesis
Jam3	3	3	C	-	C	spermatogenesis
Katnal1	7	-	-	-	D	spermatogenesis
Kit	2	4	C	MI	B	spermatogenesis
Klhdc3	5	-	-	-	E	meiosis
Klhl10	8	8	E	PM	E	spermatogenesis
Krt9	-	-	-	-	-	spermatogenesis
Lamp1	-	-	D	-	D	spermatogenesis
Lfng	1	-	-	SO	A	meiosis
Lgr4	3	2	A	SO	B	spermatogenesis
Lhcgr	2	-	-	-	E	spermatogenesis
Lif	-	-	-	SO	-	meiosis
Limk2	2	-	B	-	C	spermatogenesis
Mael	6	7	D	ME	D	meiosis, spermatogenesis
Mapk1ip1	3	-	-	-	C	meiosis
Mas1	8	8	E	PM	E	spermatogenesis
Mast2	-	-	-	-	C	spermatogenesis
Mea1	7	7	-	ME, PM	E	spermatogenesis
Mei1	3	-	-	-	B	meiosis, spermatogenesis
Meig1	7	8	D	-	D	meiosis, spermatogenesis
Mertk	3	2	-	-	B	spermatogenesis
Micalcl	8	8	E	PM	E	spermatogenesis
Mif	2	-	-	MI	A	spermatogenesis
Mki67	3	2	-	-	B	meiosis
Mkks	3	2	-	-	B	spermatogenesis
Mlh1	3	-	-	-	C	meiosis, spermatogenesis
Mlh3	4	6	D	ME	C	meiosis
Mnd1	3	3	A	-	-	meiosis
Mns1	6	6	D	ME	D	meiosis

Gene	Temporal 1-8	Schultz'03 1-8	Shima'04 A-E	Chalmel'07 SO,MI,ME,PM	Deconvolution A-E	Biological Process (GO)
Morc1	2	2	B	MI	B	spermatogenesis
Mos	-	-	-	-	-	meiosis
Mre11a	-	-	-	-	C	meiosis
Msh2	2	2	B	MI	B	meiosis
Msh3	-	6	-	-	B	meiosis
Msh4	4	6	D	PM	C	meiosis, spermatogenesis
Msh5	4	5	-	-	C	meiosis
Msh6	3	2	-	-	C	meiosis
Msx1	-	-	-	-	-	meiosis
Msx2	-	-	-	-	-	meiosis
Mtl5	6	6	D	ME	C	spermatogenesis
Mycbpap	8	-	-	PM	E	spermatogenesis
Nanos2	-	-	-	-	-	meiosis, spermatogenesis
Nanos3	1	-	-	-	A	spermatogenesis
Nbn	-	2	-	-	B	meiosis
Nek2	6	7	D	ME	C	meiosis
Nkd1	1	-	-	-	A	spermatogenesis
Nlrp14	7	8	D	ME	E	spermatogenesis
Nme5	7	7	D	ME	E	spermatogenesis
Notch1	2	2	C	SO	A	spermatogenesis
Nphp1	6	7	D	ME	D	spermatogenesis
Npm2	6	-	-	-	-	meiosis
Nr0b1	3	3	C	MI	B	spermatogenesis
Nr2c2	4	-	-	-	C	spermatogenesis
Nr6a1	5	8	-	PM	E	spermatogenesis
Odf1	8	8	E	PM	E	spermatogenesis
Odf2	7	8	D	ME	E	spermatogenesis
Odf3	8	8	E	PM	E	spermatogenesis
Odf4	7	-	-	ME	D	spermatogenesis
Osm	-	-	-	-	-	meiosis
Ovol1	7	7	-	ME	D	spermatogenesis
Pacrg	7	7	D	ME	E	spermatogenesis
Pafah1b1	-	4	B	MI	B	spermatogenesis
Pafah1b2	-	7	E	-	C	spermatogenesis
Pafah1b3	1	3	B	-	A	spermatogenesis
Pank2	-	5	D	-	D	spermatogenesis
Patz1	1	2	A	MI	A	spermatogenesis
Pax5	8	-	-	PM	E	spermatogenesis
Pcsk4	7	7	-	ME	E	spermatogenesis
Pcyt1b	2	4	-	SO	B	spermatogenesis
Pde3a	-	-	-	-	A	meiosis
Pebp1	7	-	-	-	D	spermatogenesis
Pgm3	3	3	C	MI	C	spermatogenesis
Phc2	2	-	-	-	A	spermatogenesis

Gene	Temporal 1-8	Schultz'03 1-8	Shima'04 A-E	Chalmel'07 SO,MI,ME,PM	Deconvolution A-E	Biological Process (GO)
Piwil1	6	-	-	ME	D	meiosis, spermatogenesis
Piwil2	4	5	D	ME	C	meiosis, spermatogenesis
Piwil4	2	-	-	-	A	meiosis, spermatogenesis
Plcb1	-	-	-	-	-	meiosis
Plekha1	2	2	B	MI	B	spermatogenesis
Plk1	5	7	E	ME	E	meiosis
Pms1	2	-	-	-	A	meiosis
Pms2	3	3	C	-	C	meiosis
Ppp1ca	2	-	-	MI	A	meiosis
Ppp2ca	2	2	-	MI	B	meiosis
Prdm9	3	-	-	-	B	meiosis, spermatogenesis
Prdx4	2	6	E	MI	B	spermatogenesis
Prkaca	-	5	-	-	D	spermatogenesis
Prm1	8	8	E	ME	E	spermatogenesis
Prm2	8	-	-	PM	E	spermatogenesis
Prm3	8	8	E	PM	E	spermatogenesis
Prok2	6	-	-	ME	D	spermatogenesis
Prss21	7	7	D	ME	E	spermatogenesis
Psmc3ip	6	7	D	ME	C	meiosis
Psmd13	-	5	-	-	C	meiosis
Psme4	6	6	E	ME	D	spermatogenesis
Pttg1	6	6	-	-	C	meiosis
Pvrl2	2	-	D	SO	C	spermatogenesis
Qk	3	2	B	MI	B	spermatogenesis
Racgap1	-	3	B	-	D	spermatogenesis
Rad18	4	-	C	ME	C	spermatogenesis
Rad23b	-	-	B	-	D	spermatogenesis
Rad50	3	2	B	-	B	meiosis
Rad51	3	3	-	MI	B	meiosis
Rad51c	-	5	D	ME	D	meiosis, spermatogenesis
Rara	1	-	-	-	A	spermatogenesis
Rbbp8	3	4	C	-	B	meiosis
Rbm7	-	2	C	SO	B	meiosis
Rbp4	3	3	C	-	C	spermatogenesis
Rec8	5	5	E	PM	E	meiosis, spermatogenesis
Rhbdd1	7	5	D	-	E	spermatogenesis
Rnf151	8	7	E	PM	E	spermatogenesis
Rnf17	4	-	C	ME	C	spermatogenesis
Rnf8	3	3	-	-	C	spermatogenesis
Ros1	-	-	-	-	-	spermatogenesis
Rpa1	5	5	D	-	D	meiosis
Rps6ka2	2	3	C	MI	B	meiosis
Rsph1	6	7	D	ME	D	meiosis
Rspo1	2	-	-	-	-	meiosis

Gene	Temporal 1-8	Schultz'03 1-8	Shima'04 A-E	Chalmel'07 SO,MI,ME,PM	Deconvolution A-E	Biological Process (GO)
S100a11	1	1	A	SO	A	spermatogenesis
Sbf1	-	5	-	-	C	spermatogenesis
Scmh1	4	5	D	ME	C	spermatogenesis
Sep15	-	5	B	-	B	spermatogenesis
Sept4	8	8	E	PM	E	spermatogenesis
Serpina5	3	3	D	MI	B	spermatogenesis
Sfmbt1	4	5	-	ME	C	spermatogenesis
Sgol1	3	-	-	-	B	meiosis
Sgol2	4	2	-	-	B	meiosis
Sgpl1	2	3	-	MI	B	spermatogenesis
Shbg	2	3	C	MI	B	spermatogenesis
Siah1a	4	6	-	-	C	meiosis, spermatogenesis
Sirt1	3	3	C	MI	B	spermatogenesis
Six5	1	1	-	-	A	spermatogenesis
Slc22a16	7	7	D	ME	D	spermatogenesis
Slc26a3	-	-	-	-	-	spermatogenesis
Slc26a6	2	-	-	-	C	spermatogenesis
Slc26a8	7	-	-	ME	D	meiosis, spermatogenesis
Slco4c1	-	-	-	-	-	spermatogenesis
Smc1a	2	2	A	MI	A	meiosis
Smc1b	4	-	-	ME	B	meiosis
Smc2	3	2	B	-	B	meiosis
Smc3	3	2	-	-	B	meiosis
Smc4	4	6	D	ME	C	meiosis
Smo	1	1	A	MI	A	spermatogenesis
Sod1	2	-	-	-	C	spermatogenesis
Sohlh1	1	-	-	MI	A	spermatogenesis
Sohlh2	3	5	C	MI	C	spermatogenesis
Sox17	5	7	-	ME	E	spermatogenesis
Sox3	1	-	-	MI	A	spermatogenesis
Sox8	1	3	A	-	A	spermatogenesis
Sox9	2	3	-	SO	A	spermatogenesis
Spaca1	8	-	-	PM	E	spermatogenesis
Spag16	7	7	D	ME	D	spermatogenesis
Spag6	7	7	D	ME	D	spermatogenesis
Spata16	7	-	-	ME	D	spermatogenesis
Spata18	8	8	E	PM	E	spermatogenesis
Spata19	8	8	E	PM	E	spermatogenesis
Spata20	8	8	E	-	E	spermatogenesis
Spata22	4	-	-	-	C	meiosis
Spata5	-	5	C	ME	B	spermatogenesis
Spata6	7	6	D	ME, PM	E	spermatogenesis
Spata9	8	8	E	PM	E	spermatogenesis
Spef2	6	-	-	PM	D	spermatogenesis

Gene	Temporal 1-8	Schultz'03 1-8	Shima'04 A-E	Chalmel'07 SO,MI,ME,PM	Deconvolution A-E	Biological Process (GO)
Spem1	8	-	-	PM	E	spermatogenesis
Spin1	2	2	A	MI	B	meiosis
Spink2	7	7	D	ME	D	spermatogenesis
Spire1	3	4	-	-	B	meiosis
Spire2	-	-	-	-	D	meiosis
Spo11	6	5	D	ME	C	meiosis, spermatogenesis
Stag2	2	2	A	MI	B	meiosis
Stag3	4	7	D	ME	D	meiosis
Stk11	-	-	-	-	C	spermatogenesis
Stra13	4	-	-	-	D	meiosis
Stra8	3	4	C	MI	B	meiosis, spermatogenesis
Strbp	4	-	-	ME	C	spermatogenesis
Styx	-	6	-	-	-	spermatogenesis
Svs2	-	-	-	-	-	spermatogenesis
Syce1	6	5	D	ME	D	meiosis
Syce2	5	6	E	PM	C	meiosis
Sycp1	4	4	C	ME	C	meiosis
Sycp2	4	-	-	ME	C	meiosis
Sycp3	4	-	-	ME	C	meiosis, spermatogenesis
Taf7l	3	-	-	PM	B	spermatogenesis
Tbpl1	6	7	D	ME	D	spermatogenesis
Tcp11	8	7	D	ME	E	spermatogenesis
Tdrd1	4	-	-	ME	C	meiosis, spermatogenesis
Tdrd5	6	-	-	ME	C	spermatogenesis
Tdrd6	7	7	D	-	D	spermatogenesis
Tdrd7	6	7	D	ME	D	spermatogenesis
Tdrd9	4	-	-	-	C	meiosis, spermatogenesis
Tdrkh	4	4	-	-	C	meiosis, spermatogenesis
Tex11	3	-	-	MI	B	meiosis
Tex14	-	-	-	-	B	meiosis
Tex15	3	-	-	PM	B	meiosis, spermatogenesis
Theg	8	8	E	PM	E	spermatogenesis
Tle3	1	3	B	MI	E	spermatogenesis
Tlk2	6	6	D	-	D	spermatogenesis
Tmf1	6	-	-	-	D	spermatogenesis
Tnp1	8	8	E	ME	E	spermatogenesis
Tnp2	8	8	E	PM	E	spermatogenesis
Top2a	3	3	B	-	C	meiosis
Top2b	3	2	-	MI	B	meiosis
Topbp1	3	3	-	-	C	meiosis
Trip13	3	3	-	MI	B	meiosis, spermatogenesis
Tsnax	-	-	-	-	-	spermatogenesis
Tsnaxip1	7	8	D	-	D	spermatogenesis
Tssk1	8	8	E	PM	E	spermatogenesis

Gene	Temporal 1-8	Schultz'03 1-8	Shima'04 A-E	Chalmel'07 SO,MI,ME,PM	Deconvolution A-E	Biological Process (GO)
Tssk2	8	8	E	PM	E	spermatogenesis
Tssk3	8	8	-	PM	E	spermatogenesis
Tssk4	8	-	-	PM	E	spermatogenesis
Tssk5	8	-	-	-	E	spermatogenesis
Tssk6	8	8	E	PM	E	spermatogenesis
Ttll5	6	7	-	ME	D	spermatogenesis
Tubd1	6	7	D	ME	D	spermatogenesis
Txndc2	8	-	-	PM	E	spermatogenesis
Txndc8	8	8	E	PM	E	spermatogenesis
Txnrd3	7	7	D	ME	D	spermatogenesis
Tyro3	1	2	A	SO, MI	A	spermatogenesis
Ubb	6	6	-	-	C	meiosis
Ube2b	-	3	D	-	B	meiosis, spermatogenesis
Ubr2	2	-	-	SO, MI	B	meiosis, spermatogenesis
Usp42	4	5	D	ME	C	spermatogenesis
Utp14b	4	-	-	-	D	meiosis, spermatogenesis
Wee2	-	-	-	-	-	meiosis
Wnt4	1	-	-	SO	-	meiosis
Wnt5a	1	-	A	SO, MI	A	meiosis
Xrn1	3	2	-	-	B	meiosis
Ybx2	7	7	D	ME	D	spermatogenesis
Yy1	3	-	-	MI	B	spermatogenesis
Zbtb16	1	3	C	MI	A	spermatogenesis
Zfp318	4	6	E	ME	C	meiosis
Zfp35	6	6	D	ME	D	spermatogenesis
Zfp37	8	1	-	PM	E	spermatogenesis
Zfp386	2	-	-	-	C	meiosis
Zfp39	6	-	-	ME	D	spermatogenesis
Zfp41	4	5	-	ME	C	spermatogenesis
Zfp42	6	5	-	ME	D	spermatogenesis
Zfp541	7	-	-	ME	C	spermatogenesis
Zfx	2	2	-	MI	B	spermatogenesis
Zmynd15	8	7	E	ME	E	spermatogenesis
Zpbp	7	8	D	ME	D	spermatogenesis
Zpbp2	7	7	D	ME	E	spermatogenesis
Zscan2	7	-	-	ME	C	spermatogenesis
Zscan21	5	7	D	ME	C	spermatogenesis

Table S3. Expression clusters of selected genes associated with meiosis and/or spermatogenesis (cf. Table 1). This list of genes was constructed from genes associated with either the Gene Ontology (GO) term "meiosis (GO:0007126)" or "spermatogenesis (GO:0007283)" and their children GO categories. For each gene in the list, the rightmost column indicates the term(s) associated with that gene. GO was accessed through the http://www.geneontology.org/ website.

genesymbol	Iteration, p-value								description
	1, 0.05	3, 0.05	10, 0.05	100, 0.05	1, 0.001	3, 0.001	10, 0.001	100, 0.001	
Actb	AE	ACE	ACE	ACE	A	ACE	ACE	AC	actin, beta, cytoplasmic
Brca1	AB	ABE	ABE	ABE		AB	AB	AB	breast cancer 1
Cdh1	A	A	A	A	A	A	A	A	cadherin 1
Clu	ABE	ABE	ABE	ABE	A	AB	AB	AB	clusterin
Dazl	AB	AB	AB	AB	B	B	B	B	deleted in azoospermia-like
Ddx4	ABDE	ACDE	ACD	ACD	D		D	AC	DEAD (Asp-Glu-Ala-Asp) box polypeptide 4
Dhh	A	AE	AE	AD	A	A	A	A	desert hedgehog
Dmc1	B	B	B	B	B	B	B	B	disrupted meiotic cDNA 1 homolog
Etv5	AE	AE	AE	ACE	A	A	A	A	ets variant gene 5
Gdnf	A	A	A	A	A	A	A	A	glial cell line derived neurotrophic factor
Kit	AB	AB	AB	AB		A	A	AB	c-kit
Lin28	A	A	A	A	A	A	A	A	RNA-binding protein LIN-28
Notch1	A	AC	AB	AB	A	A	A	A	Notch gene homolog 1
Pramel1	AB	ABC	AB	AB	B	A	B	B	preferentially expressed antigen in
Prdm9	B	B	B	B	B	B	B	B	Prdm9 protein.
Prm1			E	E			E	E	protamine 1
Prm2			E	E			E	E	protamine 2
Prm3	E	E	DE	DE		E	E	E	protamine 3
Psmc3ip	ACDE	ACDE	ACDE	ACDE	DE	DE	D	AC	proteasome (prosome, macropain) 26S subunit,
Rad51	AB	AB	AB	AB	B	B	B	AB	RAD51 homolog
Spo11	D	CDE	CD	C	D	C	C	C	sporulation protein, meiosis-specific, SPO11
Stk31	ABD	ACD	AC	BC	D	C	C		serine threonine kinase 31
Stra8	AB	AB	AB	AB					stimulated by retinoic acid gene 8
Sycp1	BD	BC	BC	B				B	synaptonemal complex protein 1
Sycp2	BD	CD	C	BC		C	C		synaptonemal complex protein 2
Sycp3	BD	ACD	AC	BC		C	C		synaptonemal complex protein 3
Trp53	ACE	ACE	ACDE	ACE	A	AC	A	AC	transformation related protein 53
Tuba1a	AC	AC	AB	AB		A	A	A	tubulin, alpha 1
Tuba1b	A	A	A	AC	A	A	A	A	tubulin, alpha 1B
Tuba1c	AE	AE	AE	AE	A	AE	AE	AE	tubulin, alpha 1C
Tuba3a	ADE	ADE	ADE	ACDE		ADE	AD	AD	tubulin, alpha 1a
Tuba3b	ADE	ADE	ADE	ACD	D	ADE	D	D	tubulin, alpha 7
Tuba4a	AE	ABE	ABE	ABE	E	E	AE	ABE	tubulin, alpha 4
Tuba8		E	AE	AE		E	E	E	tubulin, alpha 8
Tubb2a	A	A	A	A		A	A	A	tubulin, beta 2
Tubb2c	ACDE	ACDE	ACDE	ACDE	ADE	ADE	ADE	ACE	tubulin, beta 2c
Tubb3	AC	ACE	AC	AB					tubulin, beta 3
Tubb5	A	AE	AE	AE	A	A	A	A	tubulin, beta 5
Tubb6	AE	AE	AE	AE	A	AE	AE	A	tubulin, beta 6
Vezt	ABE	ACE	ACE	ABE					transmembrane protein vezatin

Table S4. Deconvolution classification of gene expression by cell type. For a selection of genes, we show the cell types with estimated non-zero expression, for different iterations and significance cutoffs.

Sample	6dpp	10dpp	12dpp	14dpp	16dpp	18dpp	20dpp	38dpp	Pooled
No. splices, ≥1 hits	117148	110113	105727	110120	105395	117322	102048	97937	153864
No. splices, ≥2 hits	92227	85409	79886	84523	78626	95148	75605	70262	140745

Table S5. Detection of known exon-exon splices. There are 205151 known splices, based on known gene isoforms, and about 75% of them are detected in our data. When demanding ≥2 hits, only distinct hits qualify.

Sample	6dpp	10dpp	12dpp	14dpp	16dpp	18dpp	20dpp	38dpp	Pooled
No. splices, ≥1 hits	2161	1757	1882	2215	2993	3036	2067	2416	13012
No. splices, ≥2 hits	271	235	252	300	343	575	344	503	3148

Table S6. Detection of novel splices based on known isoforms. There are 2065232 (above $2\cdot10^6$) possible splices – see text for details. We see about 13000 novel exon-exon splices in total. When demanding ≥2 hits, only distinct hits qualify.

	10dpp	16dpp	Adult
**Total peaks**	20,882	68,347	15,790
**Within 250bp of knownGene TSS**	12,666	15,014	10,772
**Overlap knownGene transcipts**	15,118	55,109	11,951
**Within 250bp of all TSS**[1]	14,504	23,618	12,100
**Within 250bp of promoters**[2]	17,064	25,281	12,370
**Overlap CAGE**[3]	15,268	31,084	13,444
**Overlap CGI**[4]	13,239	14,306	10,720
**Overlap H3K4me3**[5]	18,276	27,976	14,512

[1]TSS extracted from all_mrna table of UCSC database.

[2]reported in [47].

[3]reported by stage 4 of the FANTOM project [48].

[4]CGI – CpG islands from UCSC database.

[5]C57Bl/6J wild-type mice testes as reported in [49].

Table S7. Overlaps of Pol II peaks at three time points with various marks and genomic features.

numbers of cell types	iteration(s)	p-value	genes, same (incl. unclassified)	genes, consistent (incl. unclassified)	unclassified
within each of (5, 6, 7)	1, 3	0.05	22%	na	na
within each of (5, 6, 7)	3, 10	0.05	25%	na	na
within each of (5, 6, 7)	1, 3, 10	0.05	9%	na	na
within each of (5, 6, 7)	1, 3	0.001	22%	na	na
within each of (5, 6, 7)	3, 10	0.001	26%	na	na
within each of (5, 6, 7)	1, 3, 10	0.001	10%	na	na
5, 6	1	0.05	53%	84%	1%
5, 6	3	0.05	57%	82%	0%
5, 6	10	0.05	57%	80%	0%
5, 6	3, 10	0.05	42%	54%	0%
6, 7	1	0.05	17%	68%	1%
6, 7	3	0.05	16%	61%	0%
6, 7	10	0.05	15%	56%	0%
6, 7	3, 10	0.05	10%	21%	0%
5, 6, 7	1	0.05	14%	59%	1%
5, 6, 7	3	0.05	13%	51%	0%
5, 6, 7	10	0.05	13%	47%	0%
5, 6, 7	3, 10	0.05	9%	19%	0%
5, 6	1	0.001	75%	80%	34%
5, 6	3	0.001	68%	77%	15%
5, 6	10	0.001	67%	76%	13%
5, 6	3, 10	0.001	48%	52%	9%
6, 7	1	0.001	42%	71%	29%
6, 7	3	0.001	28%	58%	11%
6, 7	10	0.001	27%	48%	7%
6, 7	3, 10	0.001	15%	23%	5%
5, 6, 7	1	0.001	36%	60%	27%
5, 6, 7	3	0.001	23%	47%	9%
5, 6, 7	10	0.001	21%	40%	6%
5, 6, 7	3, 10	0.001	14%	20%	4%

Table S8. Consistency of our deconvolution cell type assignments: comparison of the 5, 6 and 7 cell-type calculations. In total, 14259 genes were included in deconvolution. In the first six rows, "within each of (5, 6, 7)" indicates comparisons made within calculations with identical cell types at different iterations, but for all three cell type numbers. For example, in the first row, a gene having identical expression presence in iterations 1 and 3 in 5 cell-type calculation with p-value cutoff of 0.05, and having identical (but possibly different from a 5 cell-type) expression presence in iterations 1 and 3 in 6 cell-type calculation with the same p-value cutoff, and similarly for 7 cell-type calculation, will contribute one count. The remaining rows compare all of the indicated cell type-number calculations, at one or two iterations and indicated p-value cutoffs.

5 cell types Pearson correlation

	A	B	C	D	E
1 3	0.99	0.99	0.59	0.76	0.88
1 10	0.90	0.98	0.52	0.73	0.54
3 10	0.94	1.00	0.91	0.98	0.80

min=0.52

5 cell types Spearman correlation

	A	B	C	D	E
1 3	0.96	0.74	0.44	0.83	0.86
1 10	0.93	0.72	0.34	0.77	0.83
3 10	0.97	0.88	0.82	0.92	0.90

min=0.34

6 cell types Pearson correlation

	A	Bs	Bl	C	D	E
1 3	0.99	0.72	0.73	0.65	0.69	0.86
1 10	0.99	0.80	0.76	0.54	0.80	0.55
3 10	1.00	0.97	0.95	0.83	0.96	0.82

min=0.54

6 cell types Spearman correlation

	A	Bs	Bl	C	D	E
1 3	0.96	0.43	0.48	0.52	0.84	0.87
1 10	0.95	0.12	0.33	0.36	0.78	0.84
3 10	0.98	0.25	0.66	0.65	0.92	0.91

min=0.12

5 vs. 6 cell types Pearson correlation

	A	B,Bs	B,Bl	C	D	E
1 1	1.00	0.90	0.67	0.89	0.99	1.00
1 3	0.99	0.75	0.72	0.65	0.74	0.86
1 10	0.99	0.78	0.62	0.55	0.84	0.55
3 1	0.99	0.84	0.73	0.58	0.71	0.88
3 3	1.00	0.79	0.69	0.88	0.92	1.00
3 10	1.00	0.80	0.59	0.89	0.90	0.80
10 1	0.89	0.82	0.75	0.52	0.68	0.54
10 3	0.93	0.80	0.68	0.82	0.91	0.82
10 10	0.93	0.81	0.58	0.95	0.90	1.00

min=0.52

5 vs. 6 cell types Spearman correlation

	A	B,Bs	B,Bl	C	D	E
1 1	0.99	0.63	0.46	0.81	0.95	0.96
1 3	0.96	0.44	0.54	0.53	0.83	0.86
1 10	0.95	0.28	0.6	0.36	0.77	0.83
3 1	0.96	0.41	0.46	0.44	0.84	0.87
3 3	0.99	0.32	0.71	0.78	0.96	0.96
3 10	0.98	0.28	0.73	0.77	0.91	0.93
10 1	0.93	0.42	0.42	0.34	0.78	0.84
10 3	0.96	0.26	0.68	0.65	0.91	0.88
10 10	0.98	0.29	0.76	0.85	0.93	0.95

min=0.26

Table S9. Tables of Pearson and Spearman correlation coefficients for each considered cell type, between different iterations (1, 3 and 10), as indicated in the left columns. Here we consider 5- and 6-cell type results. In the 6-cell type analysis, type B is split into Bs (types A and B spermatogonia) and Bl (pre-leptotene and leptotene spermatocytes). For 5 vs. 6 cell types tables, the first (left) iteration is for 5-cell type, and the second (right) is for 6-cell type calculations. We find a good persistence of results from iteration to iteration. Given the similar cell type fraction values for types Bs and Bl these two types are by far the least stable in the 6-cell type analysis.

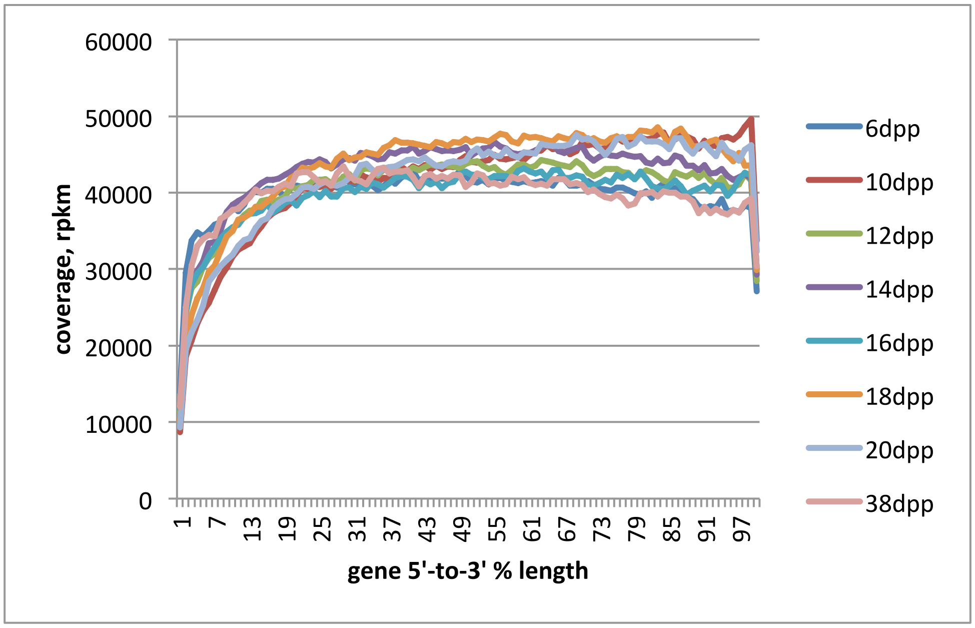

Figure S1. Read coverage along the length of expressed transcripts. Coverage is largely uniform with a slight decrease towards the 5' end.

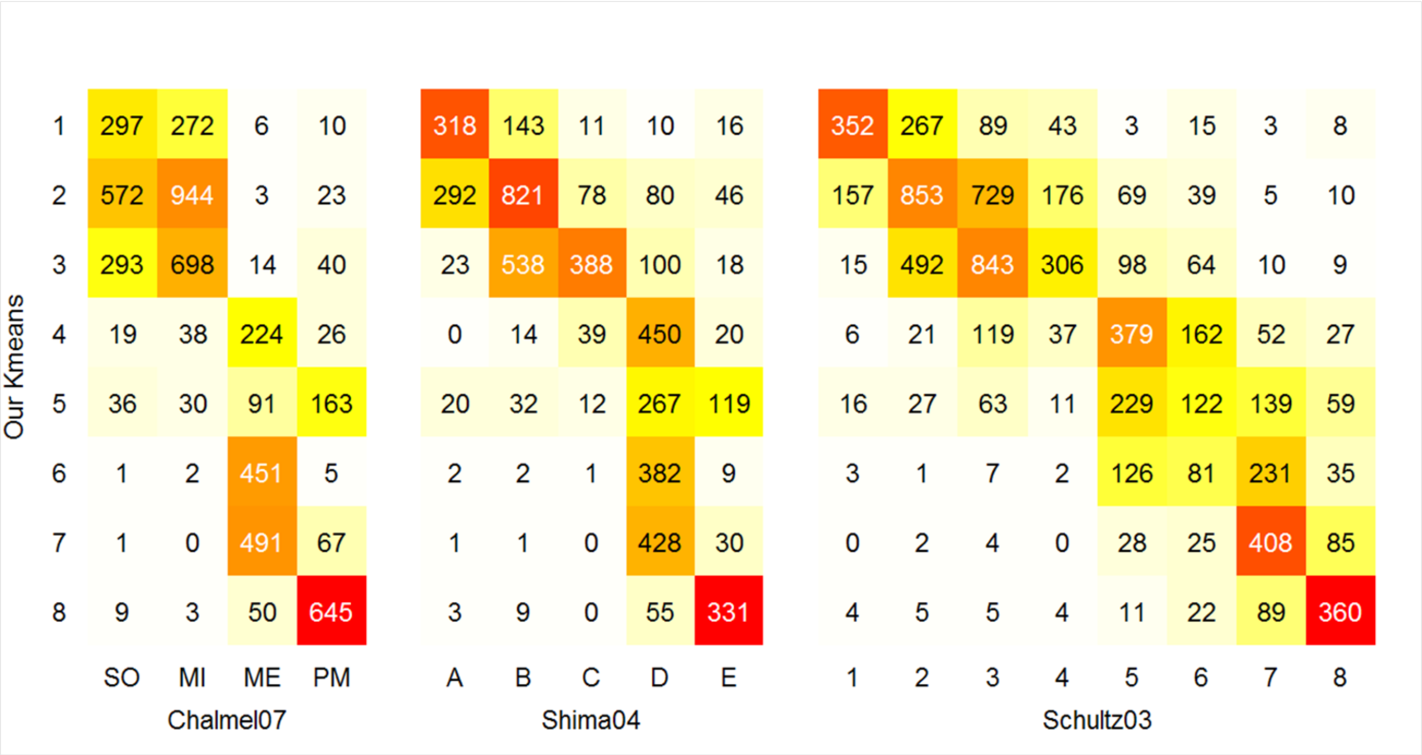

Figure S2. Comparison of our temporal expression clusters (see Figure 2) with three previously published gene expression studies [6], [8] and [9]. Chalmel et al. report 4 clusters – somatic, mitotic, meiotic and post-meiotic, while Shima et al. have 5 clusters A-E. In case of Schultz et al. we present our clustering of their microarray data. Each cell gives the number of genes common to one of our clusters and one of the clusters from the three papers. There is a reasonable agreement between various clustering results, and it is seen that our clusters 4, 6 and 7 mostly correspond to the meiotic cluster defined in Chalmel et al., to cluster D of Shima et al. and to clusters 5 and 7 derived from Schultz et al. data.

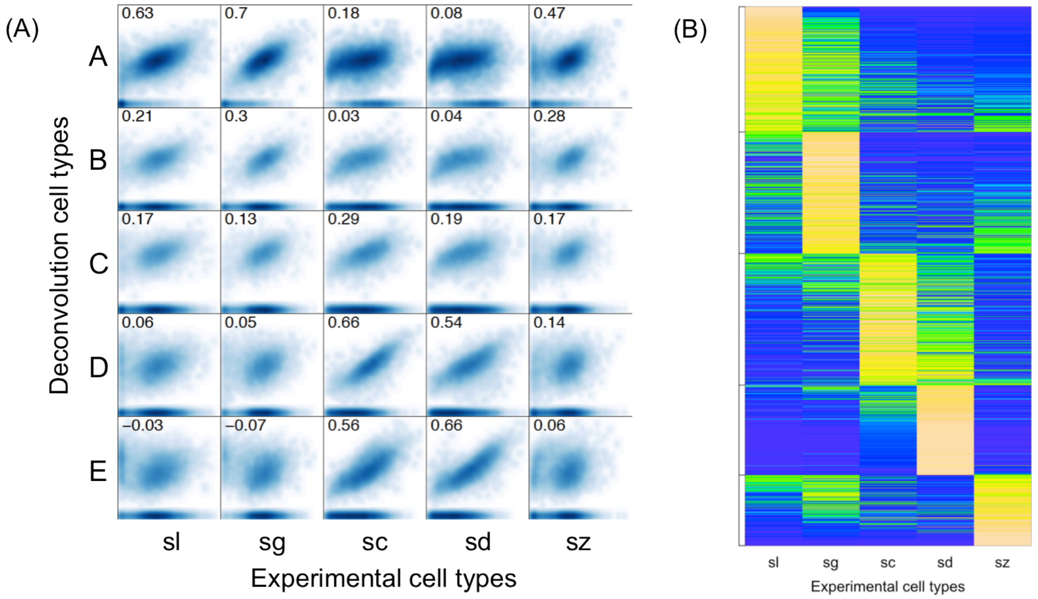

(C) Deconvolution clusters vs. Experimental clusters

	sl	sg	sc	sd	sz
A	698	877	33	11	276
B	648	1039	251	89	472
C	739	640	1020	112	357
D	116	83	1082	49	84
E	63	64	271	845	62

(D) Deconvolution clusters vs. Experimental clusters

	1	2	3	4
A	9	483	34	43
B	63	579	52	61
C	358	351	21	85
D	582	14	3	31
E	507	12	15	29

(E) Temporal clusters vs. Experimental clusters

	sl	sg	sc	sd	sz
1	330	463	14	10	99
2	864	995	63	21	426
3	574	835	98	38	392
4	93	34	647	30	72
5	94	67	354	203	57
6	3	7	485	18	8
7	4	4	389	139	5
8	10	14	33	596	15
const	328	266	572	60	174
low	251	324	67	122	309

(F) Temporal clusters vs. Experimental clusters

	1	2	3	4
1	3	210	12	27
2	17	687	44	42
3	12	447	25	69
4	192	17	1	21
5	205	7	8	21
6	283	2	0	6
7	354	2	1	7
8	283	2	3	4
const	168	39	13	40
low	254	391	1091	236

Figure S3. Comparisons of our deconvolution estimates and temporal clusters with the cell-sorted RNA-Seq data from Soumillon et al. [13]. (A) Scatter plots of log-transformed gene expression + 0.1 RPKM (a small value 0.1 is added to visualize zero expression on a log-log plot). A-E are values estimated in our respective deconvolution cell types; sl, sg, sc, sd and sz are Sertoli, spermatogonia, spermatocytes, spermatids and spermatozoa measurements from [13]. Numbers are Spearman correlation values. (B) Normalized gene expression heatmap for [13], ordered by 5 k-means clusters (our clustering of 18,411 genes from [13]). (C) Comparison of our deconvolution clusters with our clustering of data from [13] (cf. B). Each cell has the number of genes belonging to the respective deconvolution and experimental clusters. (D) Comparison of our deconvolution clusters with the four clusters defined in [13]. (E) and (F) Same as (C) and (D), but for our temporal clusters.

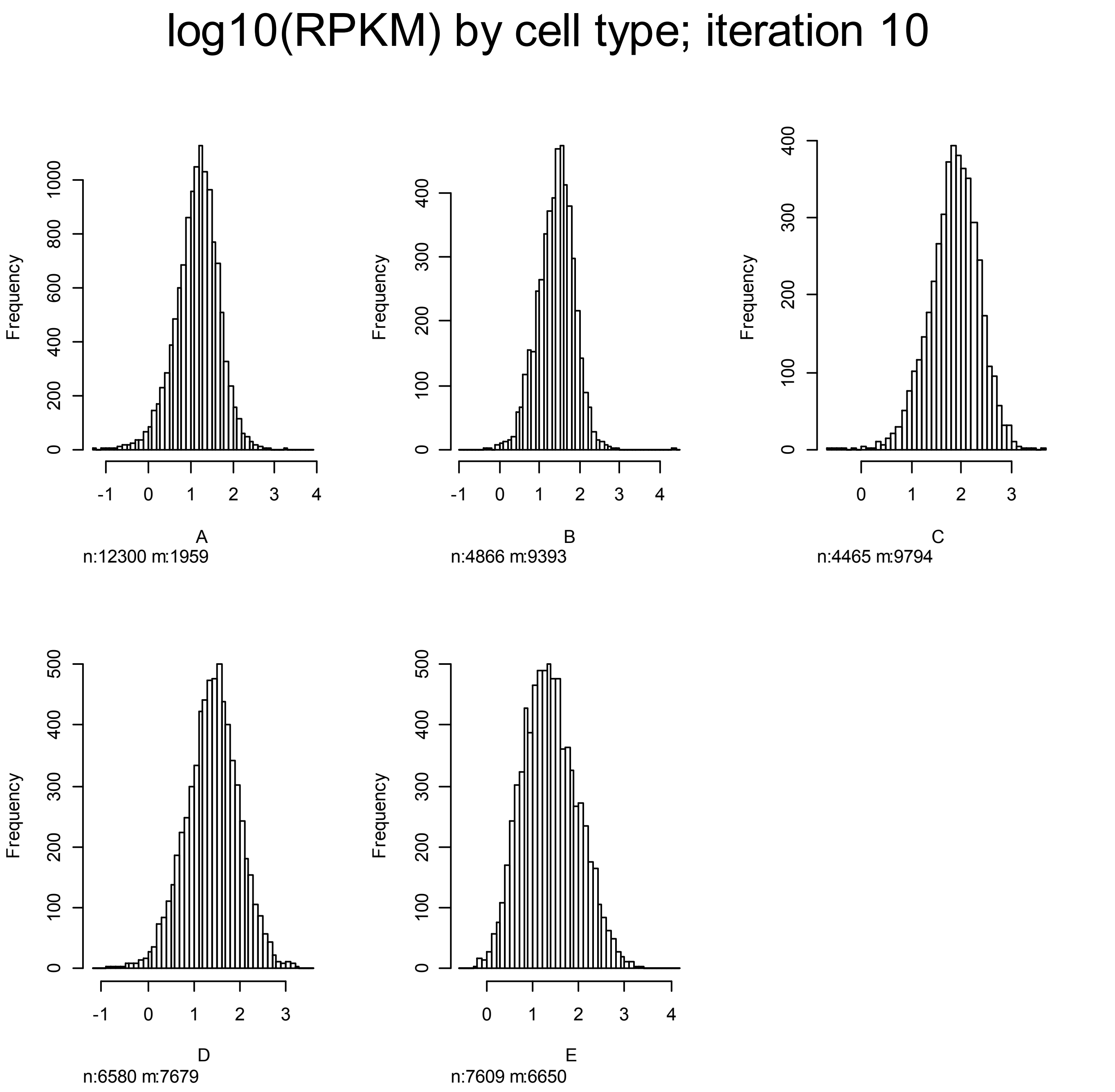

Figure S4. Histograms of predicted gene expression by cell type. n – number of genes found to be expressed in a given cell type, m – missing genes (having zero expression). Out of 14259 genes selected for deconvolution analysis, 12300 genes are predicted expressed in somatic cells (A) and 7609 are predicted to be expressed in secondary spermatocytes and/or spermatids (E). The expression signatures of other cell types are more specific and the majority of genes are predicted to be silent (or having insignificant expression) in them.

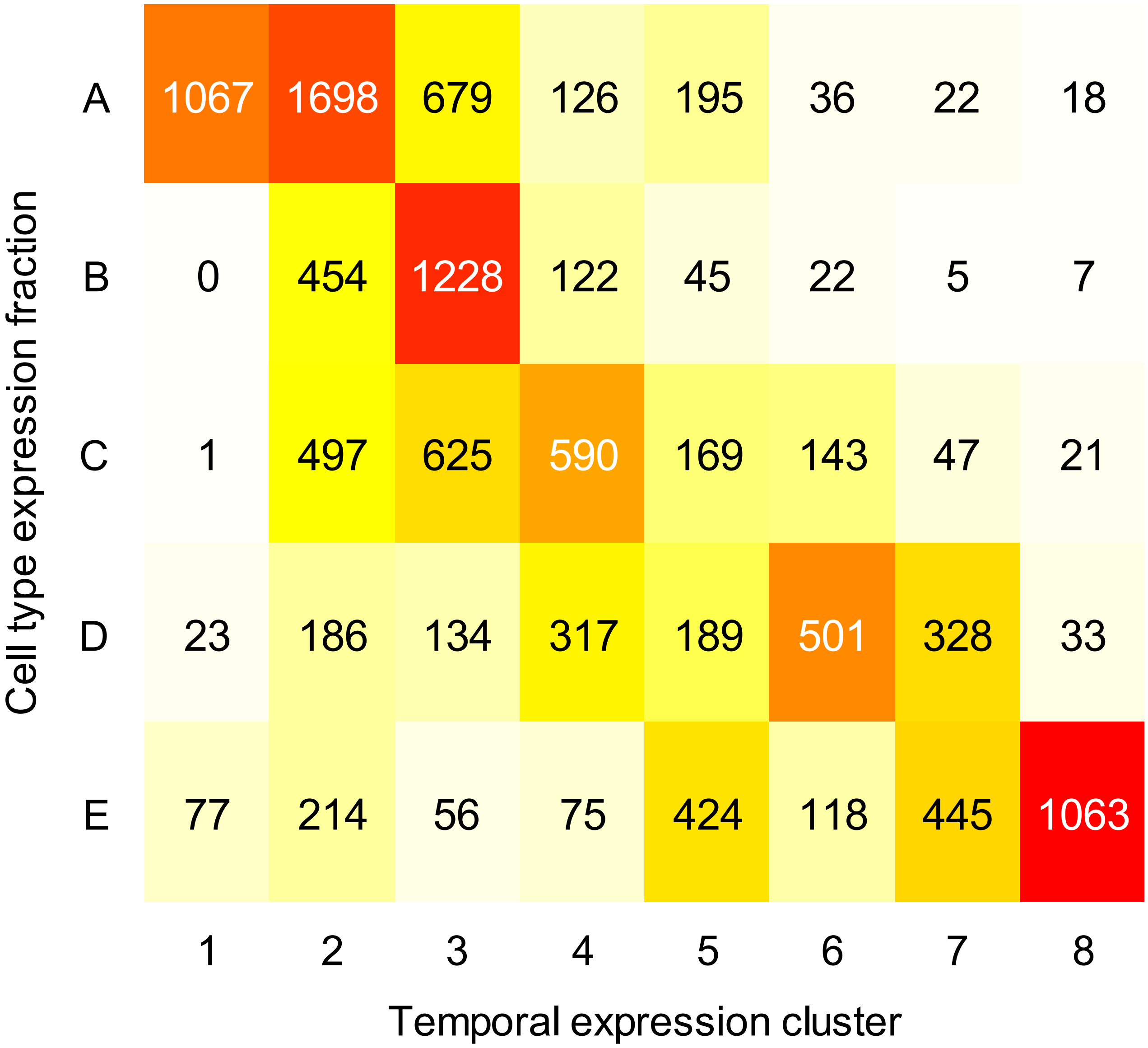

Figure S5. Comparison of our temporal clustering of gene expression with cell type-specific gene expression calculations after 10 iterations. In the deconvolution results, gene expression fraction in each cell type, A through E, is defined as the fraction of total gene expression (the sum over all cell types). Each number above is the sum of gene expression fractions for a given cell type, for genes that belong to the indicated temporal expression cluster on the abscissa. There is an overall agreement between the two analyses. In addition, a noticeable contribution to expression in post-meiotic cell type E comes from all eight temporal clusters. This indicates that there is a number of genes expressed mostly post-meiotically, but in other cell types as well, at lower levels. Given that post-meiotic cell types are practically absent until after 20dpp, such genes can fall in different temporal clusters.

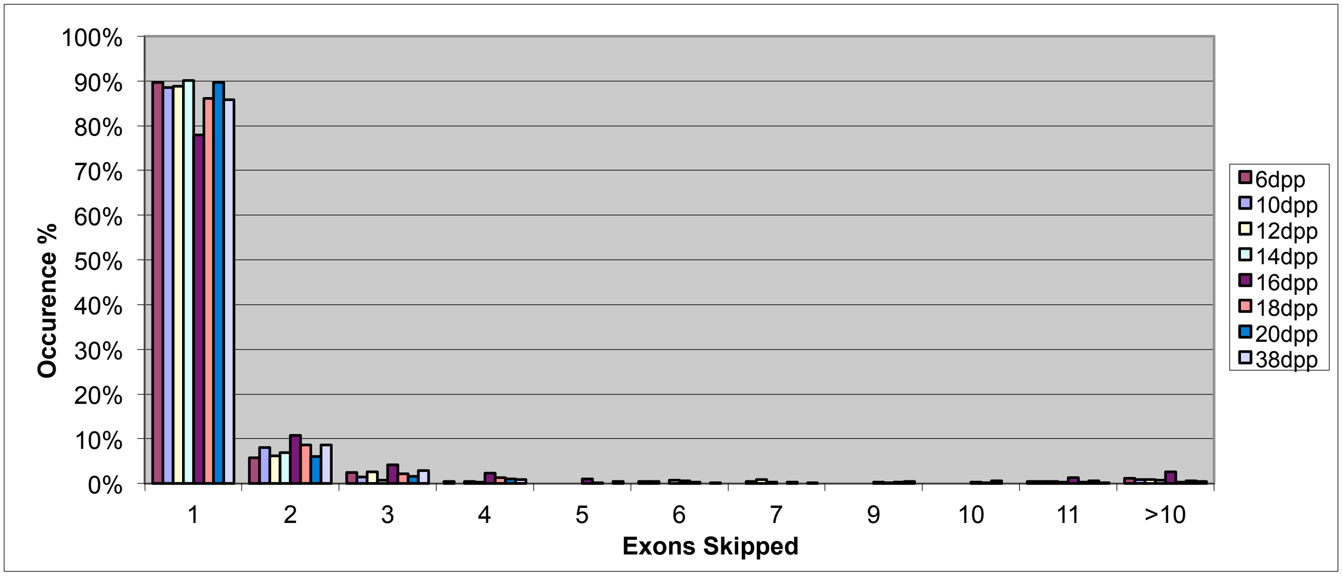

Figure S6. Distribution of the number of skipped exons in novel splices. In the case of multiple choices, we pick the smaller number. The majority (between 85 to 90%, except at 16dpp with about 77%) of novel splices have one exon skipped.

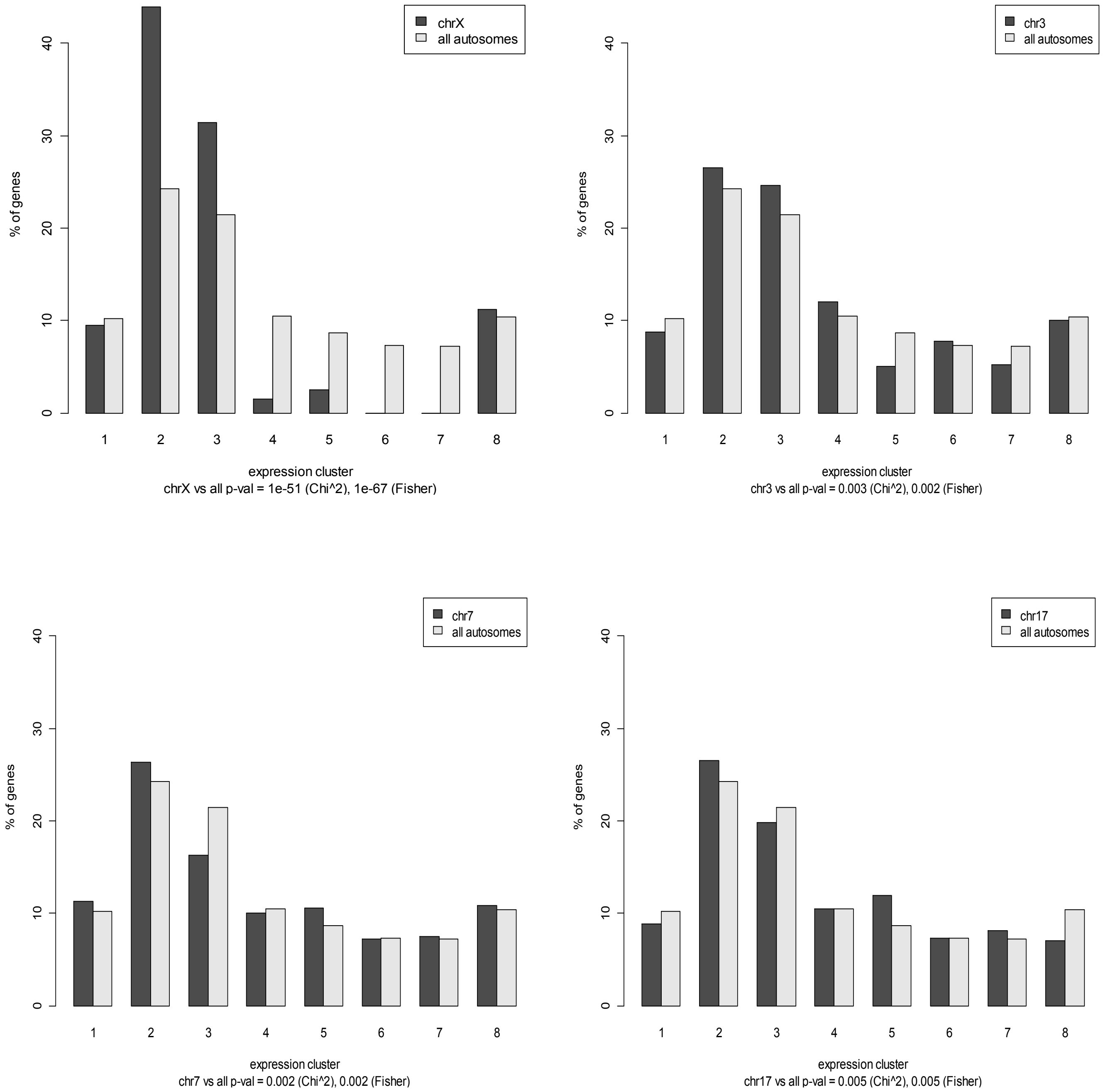

Figure S7. Chromosomes X, 3, 7 and 17 have statistically significant deviations in proportions of genes split by temporal expression cluster (black bars), when compared with all autosomes (light grey bars). Chromosome X is by far the most obvious outlier - due to MSCI, there are no X-linked genes in clusters 6 and 7, and they are strongly underrepresented in clusters 4 and 5. On the other hand, temporal clusters 2 and 3, which contain pre-meiotic and early meiotic genes (cf. Figure S), are enriched on X.

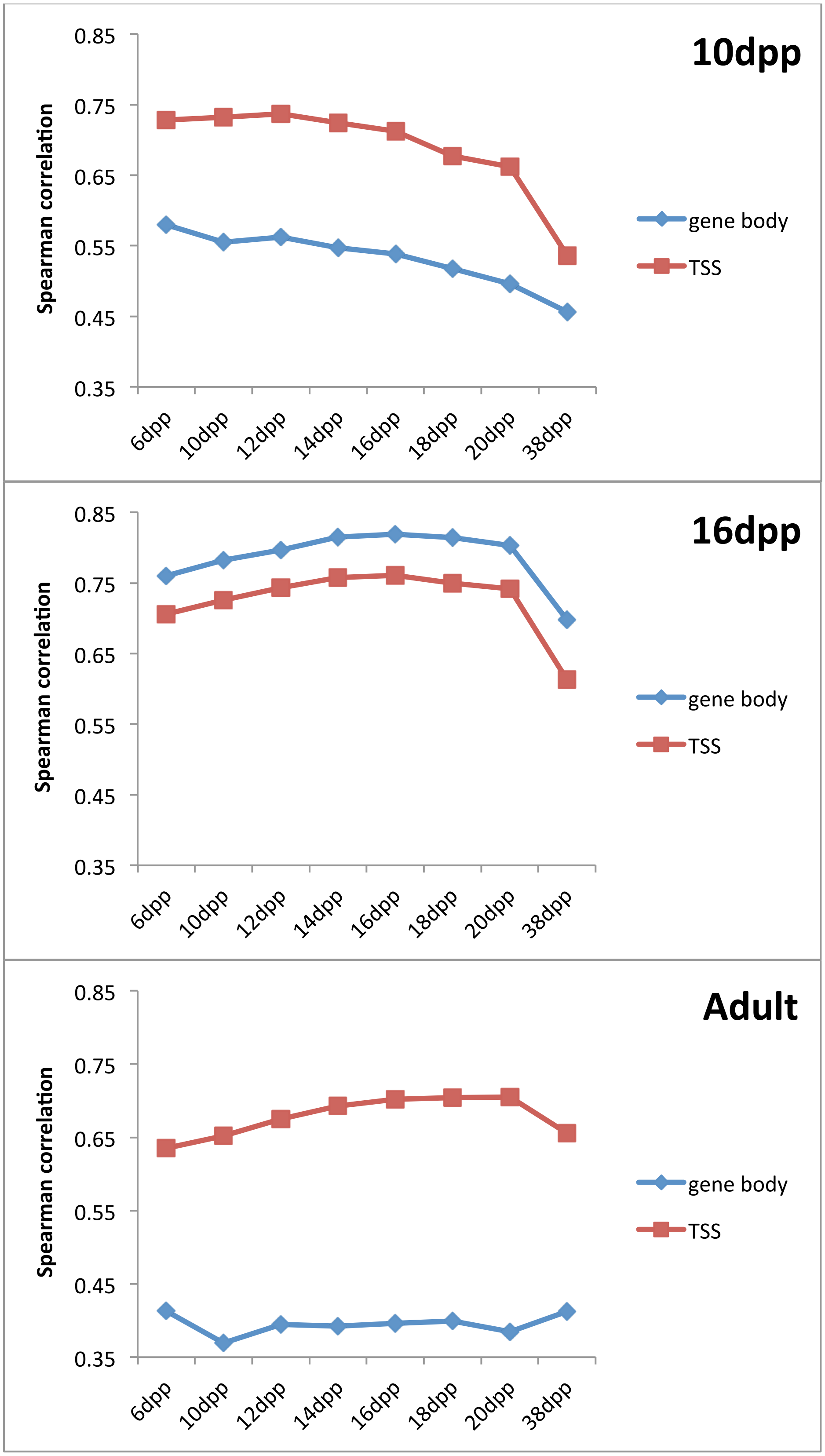

Figure S8. Spearman correlation of TSS-proximal 500bp (red) and TSS-distal/gene body (blue) Pol II read coverage with gene expression at different time points (abscissa). Pol II at 10dpp (top), Pol II at 16dpp (middle), Pol II in adult sample (bottom) are shown (See Materials and Methods for details). Gene body correlation for Pol II at 16dpp is higher than TSS-proximal correlation because in this Pol II sample we used phosphorylated Serine-5 specific antibody, which yielded more signal along transcribed genes.

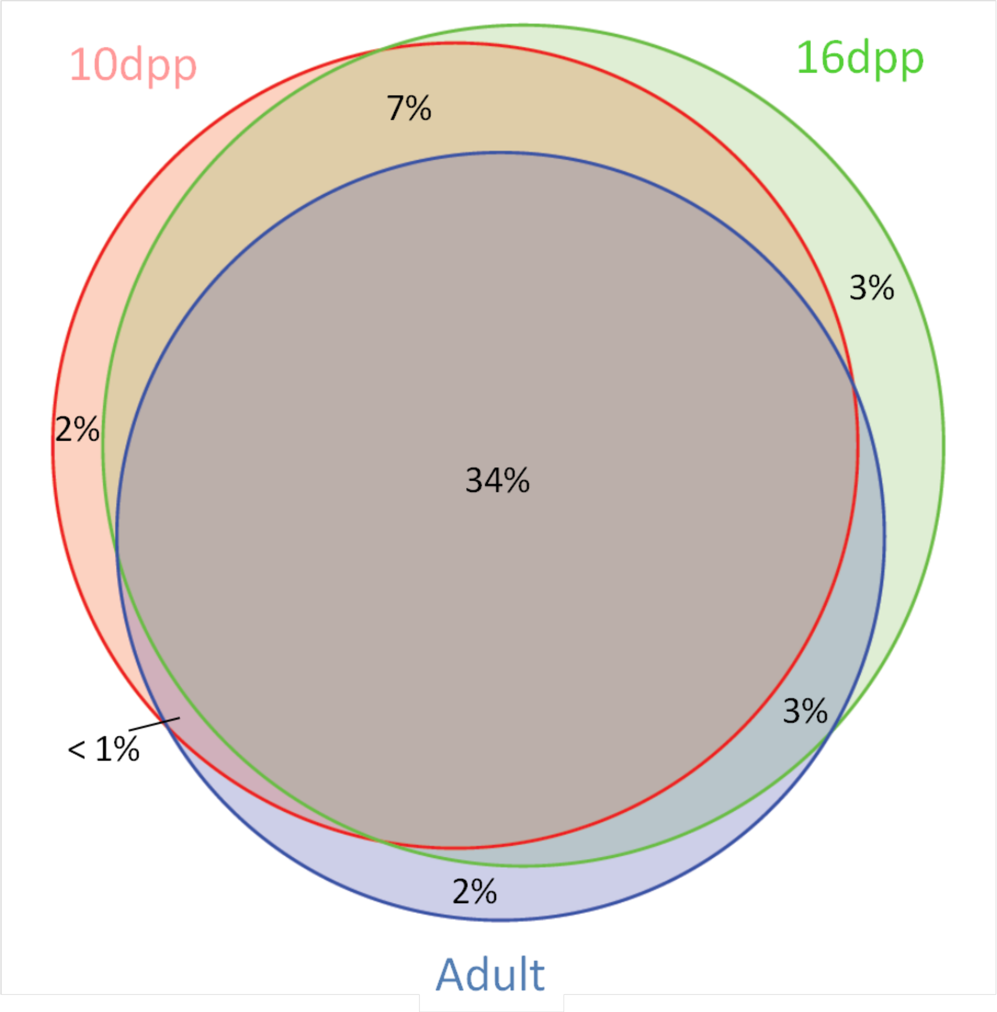

Figure S9. Venn diagram of the proportion of TSS-proximal Pol II peaks at 10dpp (red), 16dpp (green) and adult (blue) among all genes. 51% of 27,775 TSS intervals have peak at one or more time points. Note that 37% of the intervals belong to genes with low expression throughout the measured course of spermatogenesis; these genes mostly don't have Pol II peaks at their TSS's – see Figure S.

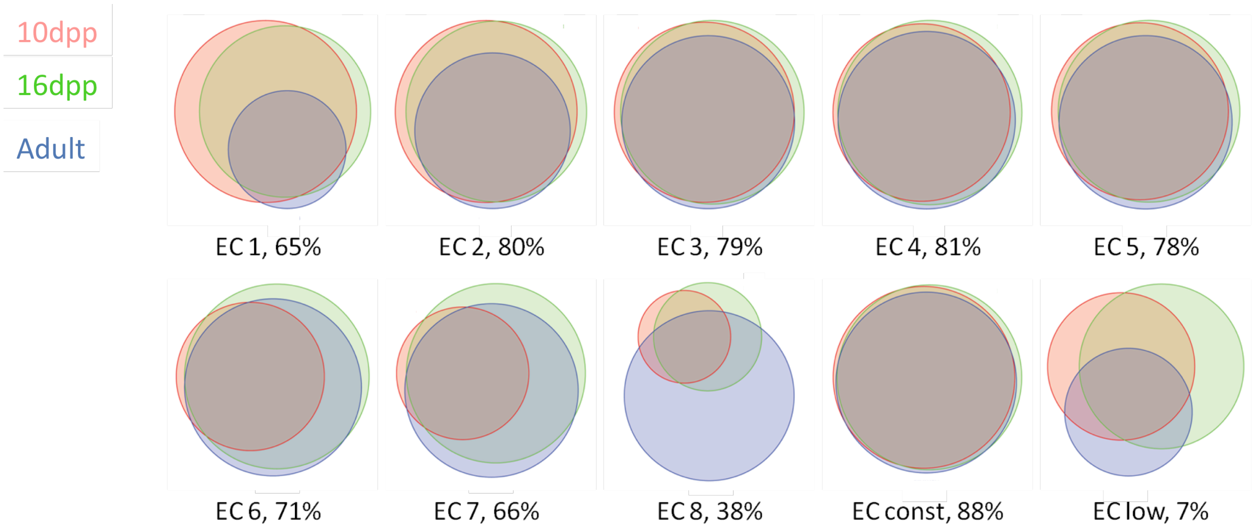

Figure S10. Venn diagrams of the proportions of TSS-proximal Pol II peaks at 10dpp (red), 16dpp (green) and adult (blue), split by gene expression cluster (EC). EC 1 through 8 are the expression clusters discussed in the paper (early to late), 'const' are genes with maximal RPKM above 2 but the fold change less than 2 over the whole time course, and 'low' are the genes with maximal RPKM below 2 at all time points. The percentage for each cluster is the percent of genes considered in that cluster that have at least one peak at any of their TSS's at any time point. For each cluster separately, circle sizes and overlaps are drawn to scale. Cluster sizes are 1215, 2875, 2437, 1031, 881, 733, 711, 1179, 1444, and 7352 for ECs 1, 2, 3, 4, 5, 6, 7, 8, 'const' and 'low', respectively.

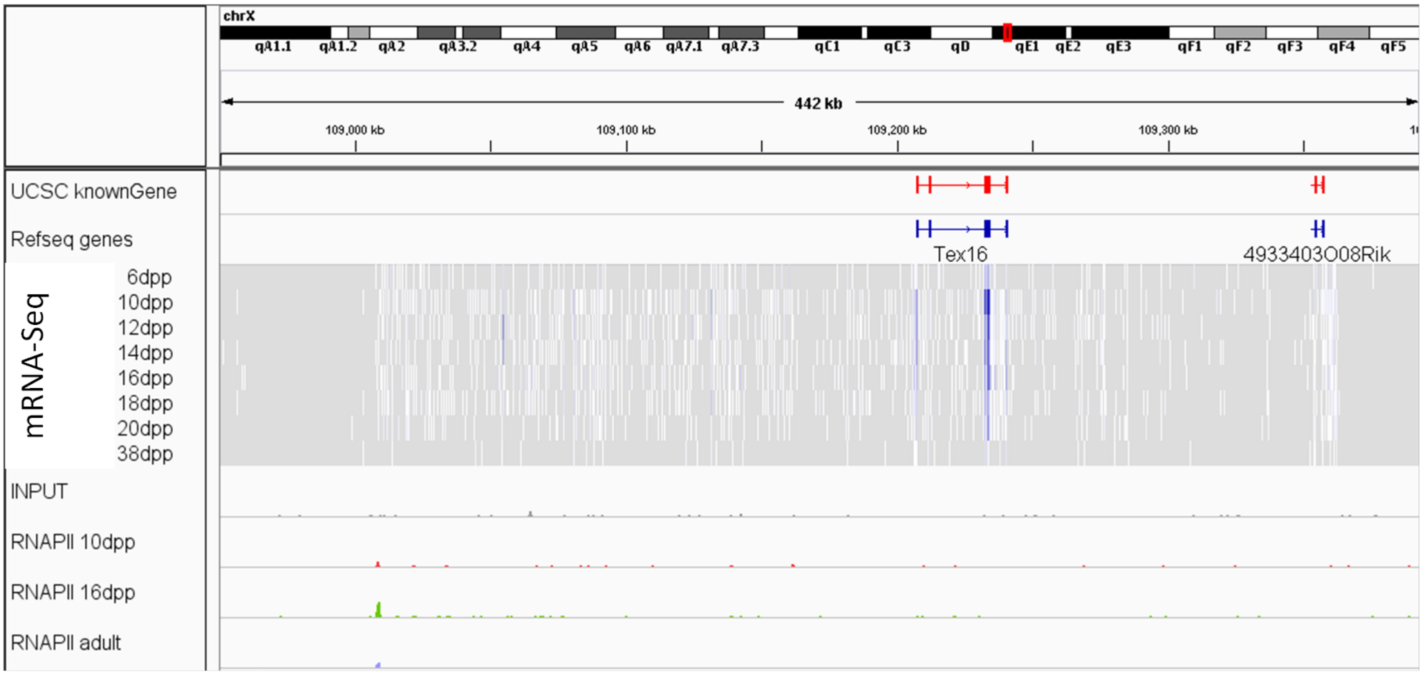

Figure S11. Annotated model of Tex16 gene possibly misses long, ~200kb 5' part. We can detect both the presence of potential exons and Pol II signal upstream of the available annotation.

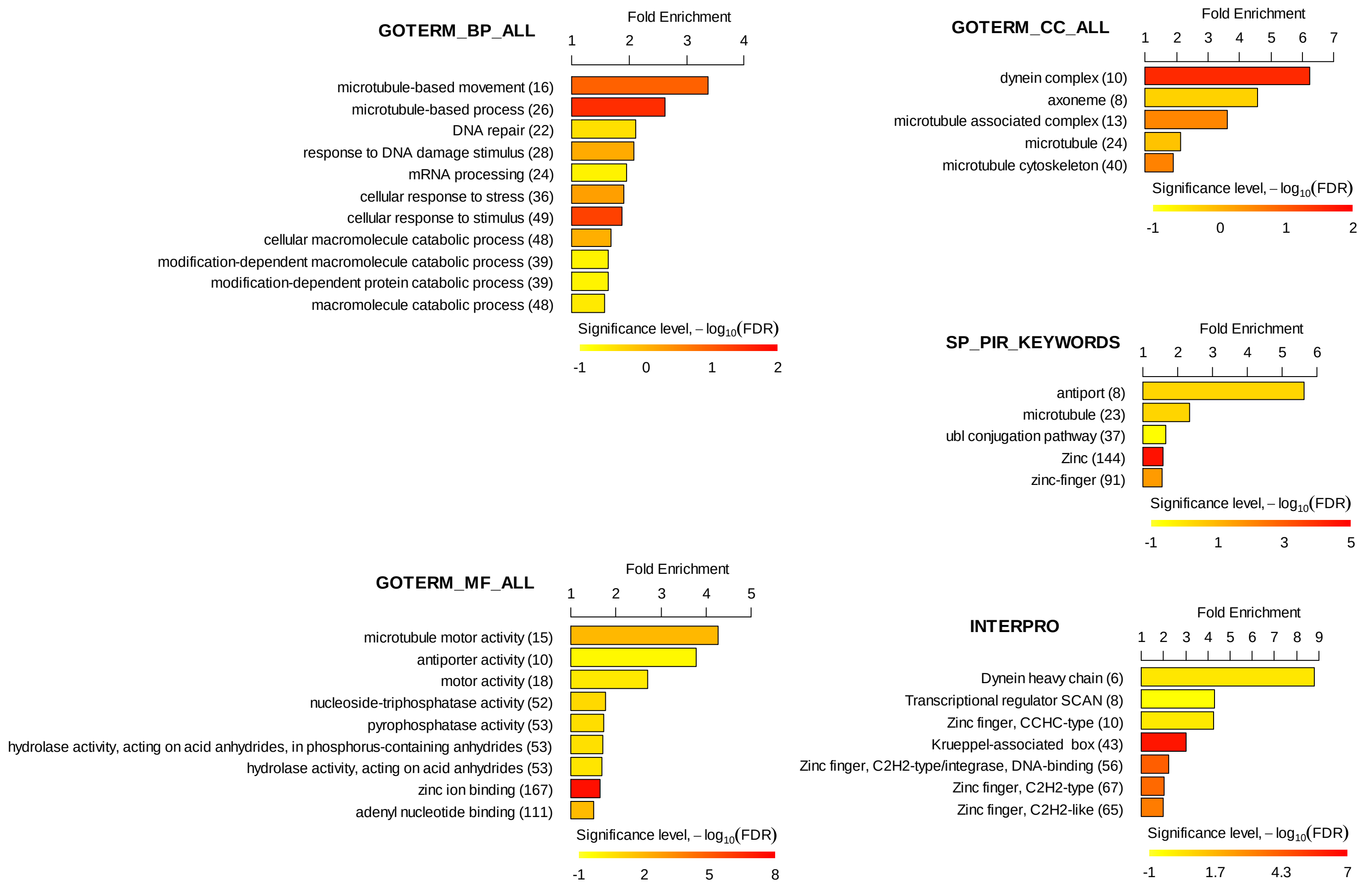

Figure S12. Enriched Gene Ontology (GO) and INTERPRO categories in novel meiotic genes in various functional annotation databases. The data is obtained from the DAVID Functional Annotation Tool [рл].

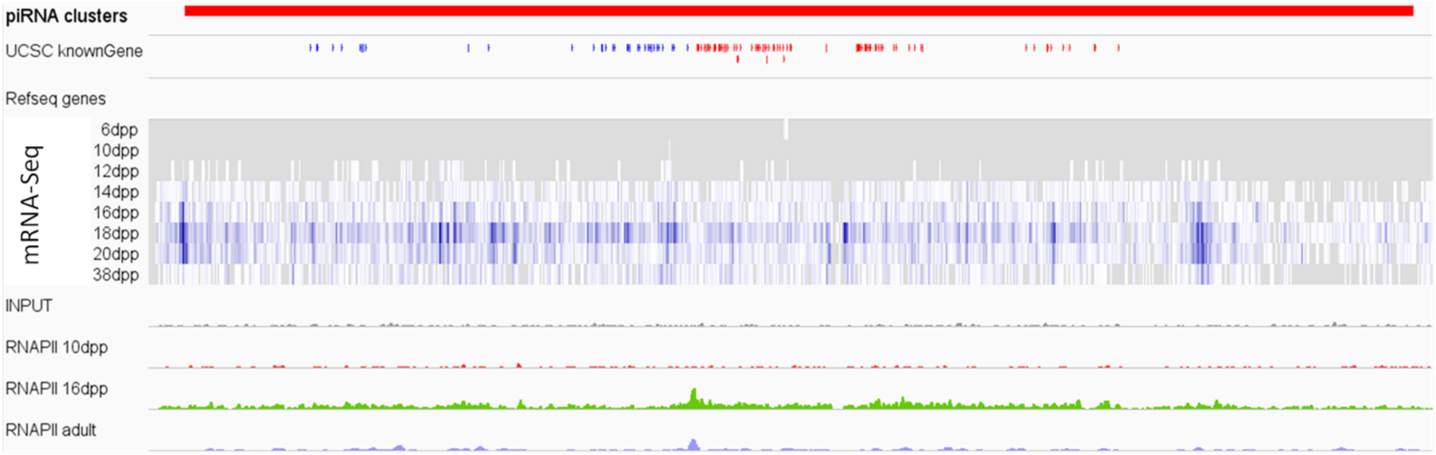

Figure S13. Example of piRNA cluster on chromosome 7. The cluster interval is based on [55] (red bar), and its coordinates are chr7:77,023,159-77,099,158. There is no RefSeq annotation in this region, however there are multiple short gene transcripts annotated in UCSC knownGene. Short gene transcripts on the forward and reverse strand are shown in red and blue colors, respectively. mRNA signal starts to be noticeable at around 14dpp, and there is a corresponding Pol II signal at 16dpp. There is no Pol II signal at 10dpp, and there is a weaker signal in the adult sample. Note that in this and some other examples there is a distinguishable Pol II peak marking the divergent transcription exactly in agreement with the UCSC annotation.

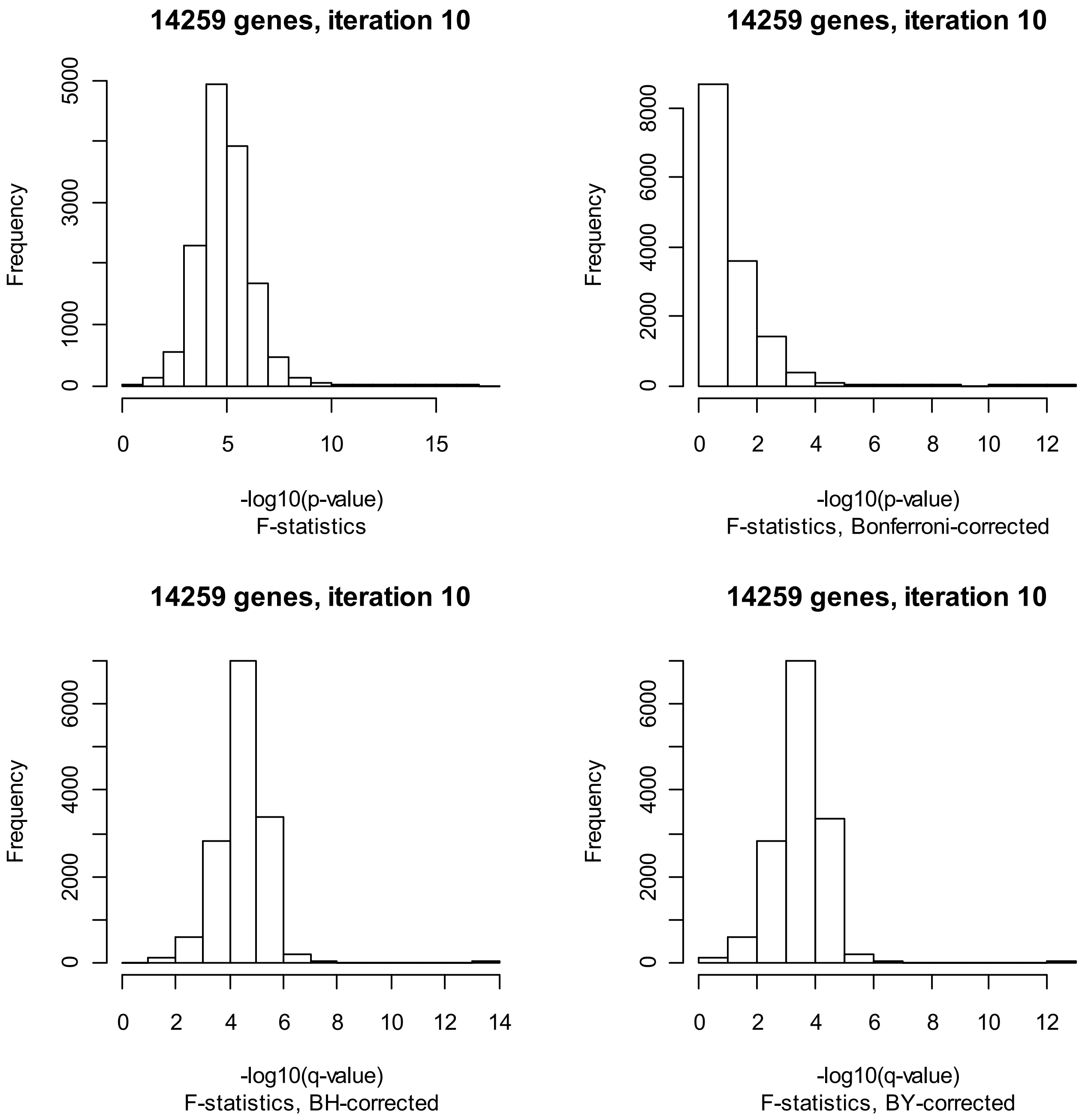

Figure S14. Initial and corrected significance values of F-statistics (testing for non-randomness of deconvolution algorithm predictions). BH – Benjamini-Hochberg, BY – Benjamini-Yekutieli procedures. We note that these estimates do not take the stochastic nature of our algorithm into account. Nevertheless, they should provide certain confidence in the statistical significance of our results.

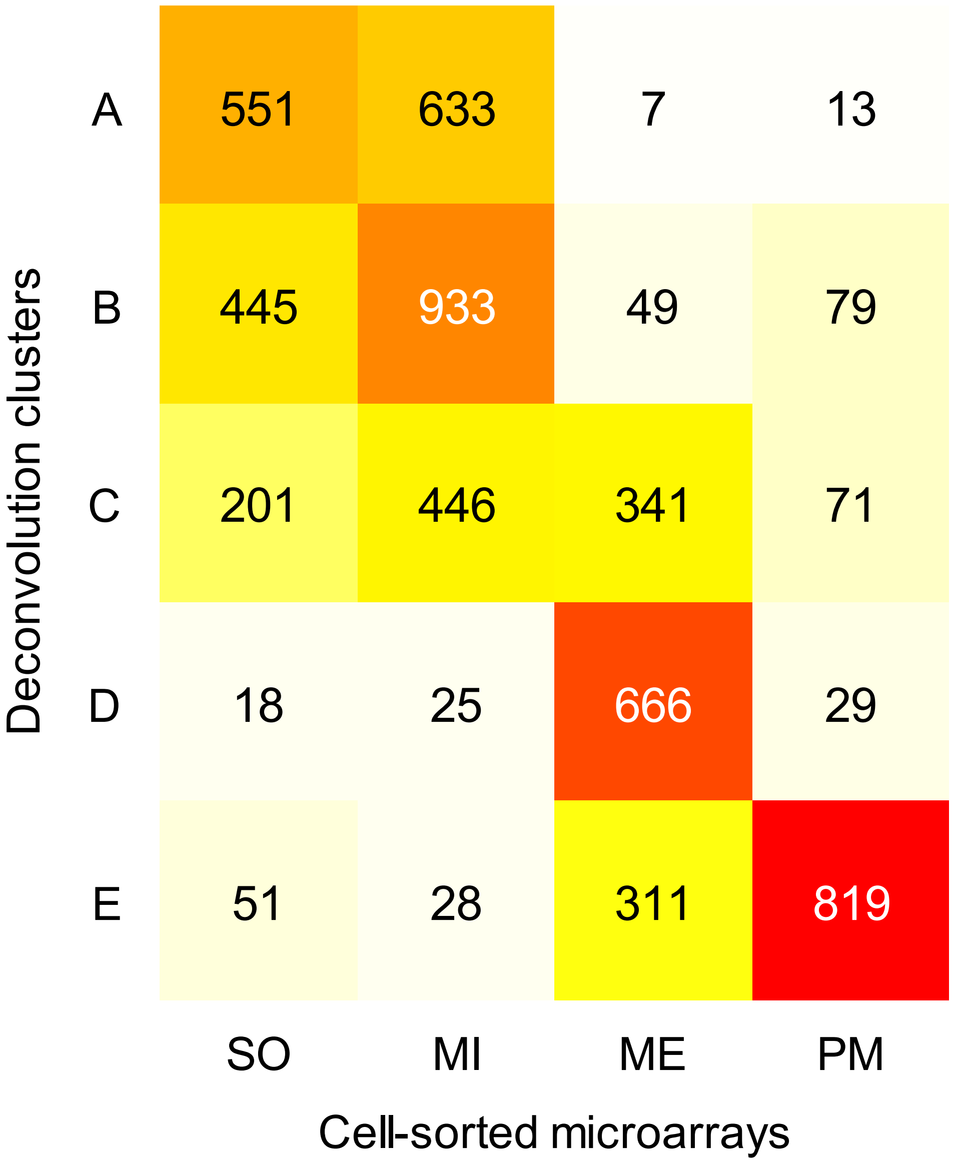

Figure S15. Comparison of genes predicted by our deconvolution analysis to lie in clusters corresponding to cell types A-E (after 10 iterations; cf. Figure 4) with experimental cell type sorted results of Chalmel et al. [6]. SO, MI, ME, PM stand for somatic, mitotic, meiotic and post-meiotic clusters.

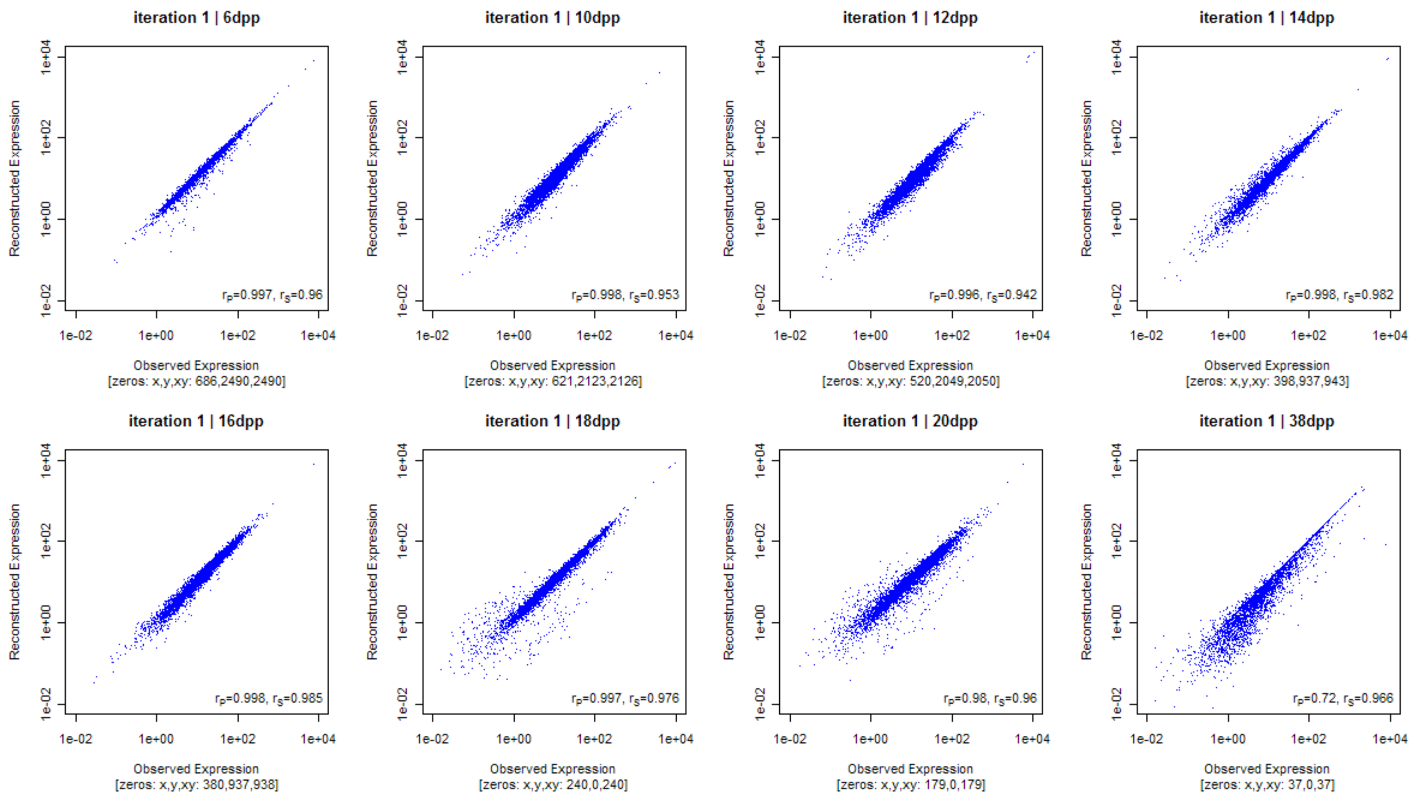

Figure S16. Comparison of observed and reconstructed gene expression datasets after 1 iteration (i.e., with the initial estimate of cell type fractions). Pearson and Spearman correlation coefficients are given. Numbers of genes with zero expression for observed, reconstructed and both datasets are provided in square brackets as well. Reconstructed temporal gene expression is based on cell type contributions at different dpp, and on the estimated cell type-specific gene expressions.

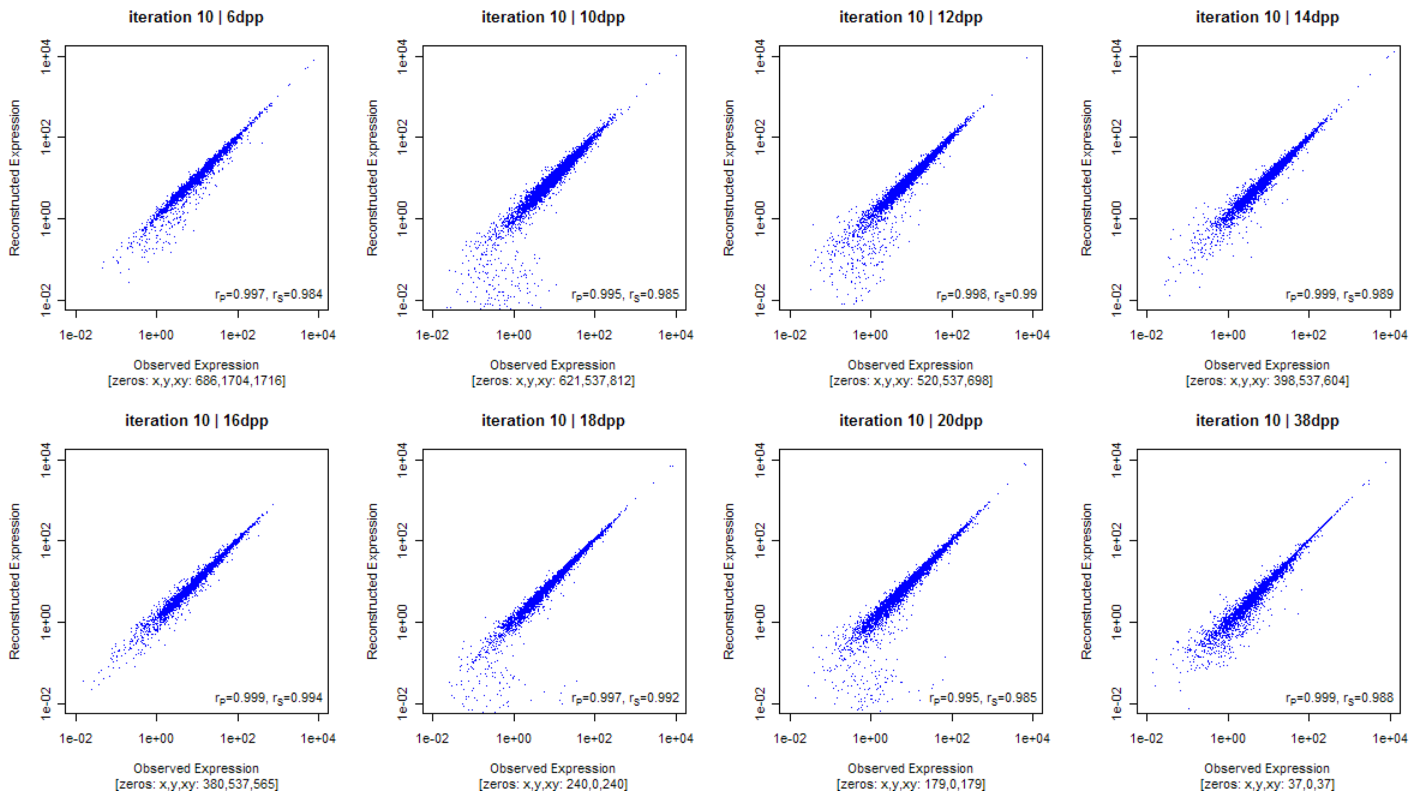

Figure S17. Comparison of observed and reconstructed gene expression datasets after 10 iterations. Pearson and Spearman correlation coefficients are given. Numbers of genes with zero expression for observed, reconstructed and both datasets are provided in square brackets as well. In comparison to Figure S16 for iteration 1, a better agreement is found here, especially at 38dpp.
